# Supplementary material for: Protein sequence design with a learned potential
Source: Nat Commun. 2022 Feb 8;13:746. doi: 10.1038/s41467-022-28313-9 (PMC8826426; doi:10.1038/s41467-022-28313-9)
Supplement: Supplementary file 1 — Supplementary Information [file 41467_2022_28313_MOESM1_ESM.pdf]

Supplementary Information

Supplementary Note 1

Supplementary References

Supplementary Figs. 1 to 21

Supplementary Tables 1 to 10

Supplementary Note 1

Classifier performance For the conditional residue prediction task, our classifier gives an improvement of 14.7% over (*1*) (42.5%), and similar performance to (*2*) (52.4%), (*3*) (56.4%) and (*4*) (58.0%). We note that we do not use the same train/test sets as these studies. Another study uses a similar 3D CNN model for ΔΔG prediction (*5*). The baseline model has a 33.5% test set accuracy. The model often confuses large hydrophobic residues phenylalanine (F), tyrosine (Y), and tryptophan (W), and within this group, more often confuses Y for F which are similarly sized compared to the bulkier W. Looking at residue-specific accuracies (Supplementary Fig. 1C, top), on the whole, the conditional model is more certain about hydrophobic/non-polar residue prediction compared to hydrophilic/polar. Both the conditional and baseline models do especially well at predicting glycine and proline residues, both of which are associated with distinct backbone torsion distributions (Supplementary Fig. 1C). Lower accuracy for predicting glutamine in particular is likely a function of lower glutamine abundance as well as inherent difficulty in predicting surface polar residues without sufficient context. This could be combated in future with resampling or class weighting during training.

The conditional model is also trained to predict binned rotamer torsion (χ) angles in an autoregressive fashion. Across 24 bins, the test set accuracy of the model to within less than 15 degrees is 52.7%, 43.6%, 26.8%, and 30.6% for χ^1-χ^4, respectively (Supplementary Fig. 1D). Unlike rotamer scoring methods (*6, 7*), where the network is trained to score contextually correct rotamers from others, this model is simply trained to predict all the rotamer angles autoregressively, conditioned on context.

Generalization to unseen topologies – design baselines We assess the performance of our sequence design method by comparison with two Rosetta baselines. The first,

Rosetta-FixBB, is a fixed-backbone Rosetta design protocol, and the second, *Rosetta-RelaxBB*, interleaves backbone relaxes between fixed-backbone design cycles, allowing the template backbone to move. The *Rosetta-FixBB* protocol provides the most relevant comparison to our method as both operate on fixed backbones. However, *Rosetta-RelaxBB* is the more commonly used mode of sequence design, generating solutions that are more variable than those of *Rosetta-FixBB*. We have therefore included designs from both methods for comparison.

On average, Rosetta is more sensitive to the backbone than the model for rotamer repacking: for the test case *1acf*, Rosetta does well once the crystal structure backbone has been relaxed under the Rosetta energy function with the native sequence and rotamers in place; our model, however, is robust to the deviations in the backbone and performs similarly in both cases (Supplementary Fig. 3A,B).

The model recapitulates the native sequence to a similar extent as *Rosetta-FixBB* and to a better extent than *Rosetta-RelBB* (Supplementary Fig. 3C). While the *Rosetta-FixBB* designs are convergent, the model designs are more variable in terms of inter-sequence percent overlaps across designs (Supplementary Fig. 4B). However, both the model designs and Rosetta designs converge to similar core (low solvent-accessible surface area) regions, indicating that perhaps the need for a well-packed core in combination with volume constraints determined by the backbone topology might limit the number of feasible core designs.

Model-designed sequences adhere to an amino acid distribution similar to the native sequence and its aligned homologs (Supplementary Fig. 4A), with the most pronounced difference being an apparent overuse of lysine (K) and glutamate (E) and under-use of rarer residues (H, N, Q, W, C) relative to homologous sequences; *Rosetta-FixBB* designs also overuse K and E and under-use H, W, N, and C relative to the native MSA distribution.

Like the model, Rosetta protocols match the native distribution well, with a tendency to overuse small hydrophobic residues, in particular alanine (A), valine (V), and isoleucine (I).

Sequences designed onto the test case backbones should ideally have the following key biochemical properties (*8, 9*): (1) core regions should be tightly packed with hydrophobic residues, a feature that is important for driving protein folding and maintaining fold stability (*10–12*); (2) designs should have few exposed hydrophobic residues, as these incur an entropic penalty as a result of solvent ordering under the hydrophobic effect and an enthalpic penalty from disrupting hydrogen bonding between polar solvent molecules (*13–15*), making it energetically favorable to place polar residues at solvent-accessible positions and apolar residues at buried positions; (3) If polar residues are designed in core regions, they should be supported by hydrogen bonding networks (*16, 17*).

We compare our designs to the *Rosetta-FixBB* and *Rosetta-RelaxBB* protocol designs, as well as the native idealized structure, across key metrics (Supplementary Fig. 7). Since the designs have less than 50% sequence identity to the native on average, we also include data for the native sequence with either 50% or 75% of the residues randomly mutated; these perturbed sequences serve as negative controls and indicate expected performance for likely non-optimal deviations from the native sequence.

We also report performance across these metrics for designed sequences relaxed with the *RosettaRelax* protocol. This procedure allows the backbone and rotamers to move to adopt a lower Rosetta energy conformation. Relaxing the designs allows us to assess whether alternate rotamers and deviations of the starting protein backbone can lead to an improvement in performance across the metrics considered.

(1) *Packstat.* Model designs tend to have a lower packstat score compared to to the native sequence and the Rosetta designs (Supplementary Fig. 7A); however, on average the

packstat scores are still higher relative to random perturbations of the native sequence. While this trend seems to indicate that the model designs are less optimal than the Rosetta designs, when we look at the model designs post-relax (Supplementary Fig. 7B), the packstat scores improve and better match those of the native sequence and Rosetta designs, while the perturbed sequence packstat scores remain low. At the same time, the post-relax alpha-carbon backbone RMSDs between design methods are also comparable (Supplementary Fig. 8E). These results suggest that the designed sequences do tightly pack core regions, as slight movements of the backbone and repacked rotamers for model designs give well-packed structures.

(2) *Exposed hydrophobics.* Model designs in general tend not to have hydrophobic residues in solvent-exposed positions, similar to the Rosetta designs (Supplementary Fig. 7C). This trend in the model designs is largely due to the relative abundance of cytosolic proteins in the PDB compared to membrane proteins, which would in general have hydrophobic regions exposed to the membrane lipid environment. The native sequence for test case *3mx7* has many exposed hydrophobic residues, suggesting that the native protein might bind to a target, forming a hydrophobic interface.

(3) *Buried unsatisfied backbone atoms.* For all of the test cases except *1bkr*, model designs have similar or fewer unsatisfied backbone polar atoms compared to the native sequence (Supplementary Fig. 7D-E). For *1bkr*, although the average number of unsatisfied backbone polar atoms is greater than that of the native sequence or Rosetta designs, the distribution is fairly wide, indicating that there are many designs that have fewer unsatisfied backbone polar atoms compared to the native sequence. However, some of the 50% perturbed sequences have fewer unsatisfied backbone polar atoms than the native, suggesting that this metric alone is not sufficient for selecting or rejecting

designs.

(4) *Buried unsatisfied side-chain atoms.* Model designs across test cases have few unsatisfied buried side-chain polar atoms, similar to the native sequences and Rosetta designs (Supplementary Fig. 7F,G). This indicates that over the course of sampling, side-chains that are placed in buried regions are adequately supported by backbone hydrogen bonds or by design of other side-chains that support the buried residue.

We use Rosetta AbInitio (*18*) and trRosetta (*19*) structure prediction to assess the recoverability of native test case backbones. Selected model designs and the top Rosetta-FixBB design (best of 50 designs ranked by Rosetta energy) are recoverable by both methods, as are some of the top Rosetta-RelBB designs (best of 50 designs ranked by Rosetta energy) (Supplementary Figs. 9, 10). The 50% perturbed negative control (best of 50 designs ranked by Rosetta energy) sequences are not recoverable under Rosetta, but seem to be recoverable under trRosetta. This is likely due to the fact that native homologs can be found via multiple sequence alignment MSA) despite the sequence perturbation, and the distance and orientation distribution prediction network, having been trained on native structures, can extrapolate from the sequence coevolution signal that is retained. Still, the model designs are better recovered in terms of alpha-carbon RMSD (Å) and GDTMM score than the 50% perturbed sequence.

Heuristic structure energy: Although the Rosetta energy is not optimized during our design procedure, many of the designs have low Rosetta energy relative to perturbed sequence baselines (Supplementary Fig. 8A). After relaxing the model designs, their Rosetta energy decreases to match the native backbone, with relatively low subsequent backbone deviation; in contrast, sequences that are 50% similar to native, with random perturbations, have a high Rosetta energy, even after relax (Supplementary Fig. 8B) The model energy

too is similarly low for the model designs and the Rosetta designs, indicating that the model energy as a heuristic roughly matches Rosetta in differentiating low-energy vs. high-energy designs (Supplementary Fig. 8D). However, the model does predict clashing side-chains at times, leading to higher Rosetta scores for designs. Overall, the alpha-carbon RMSD distributions post-RosettaRelax for the model designs correspond with the native backbone relaxed distributions (Supplementary Fig. 8E).

Crystal structure data – F2 3 of the 4 symmetric subunits for F2C fold with close correspondence to the backbone template (0.88 alpha carbon RMSD). The C-terminal helix is displaced in the last of the four internal repeats. We crystallized the same F2 construct with an N-terminal His-TEV tag (F2N) instead of a C-terminal His tag (F2C) and see a similar outcome – the C-terminal helix is displaced by the His tag in either case (Supplementary Fig. 18A). In each case, the remainder of the TIM barrel folds as expected, with structural elements including loops corresponding well to the design template.

The crystal structure for F2N (1.58 Å) shows that the majority of the structure folds as expected. Higher B-factors for F2N could be due to crystal anisotropy, with a drop in quality of the diffraction for F2N after a 90 degree rotation in phi (Supplementary Fig. 18B). The N-terminal His-TEV tag is partially resolved and might help displace the C-terminal helix in the crystal (Supplementary Fig. 18C). We also note that design of Ala3 (and symmetric positions Ala49, Ala95, Ala141) seems to reduce the packing density in the core protein, unlike in F15 and sTIM11 structure *5bvl*, which have leucines designed at these positions. While the design of alanines at these positions still produces a stable design, we hypothesize that the the terminal element could be destabilized sufficiently to allow alternative arrangements of the secondary structure in the F2N crystal condition.

For F2C, we see crystal contacts between the C-terminal helix and an adjacent monomer in the crystal (Supplementary Fig. 18D). F2N is clearly monomeric post ion exchange (Fig. 16), and we believe F2C too is monomeric, with cross-monomer crystal contacts seen likely not occurring in solution. The structure shows binding of a SO4 group which coordinates His 186 and 188 on the His tag (Supplementary Fig. 18E). This interaction might also help displace the C-terminal helix from folding against the barrel for F2C, although the SO4 group binding might occur as a result of the helix displacement. As with F2N, we hypothesize that the design of Ala3 (and symmetric positions Ala49, Ala95, Ala141) might play a contributing role in the displacement of the C-terminal helix.

In summary, F2 folds to the designed TIM-barrel backbone, although the crystal structures show displacement of the short C-terminal helix; however, the remainder of the four-fold symmetric structure folds as expected.

Crystal structure data – F15C The crystal structure for F15C has a tetrameric asymmetric unit. The monomers fold to the target structure (Supplementary Fig. 19), with two of the four monomers in the asymmetric unit for 15C corresponding extremely well to the design model (monomers A, D) (0.879, 0.97 A alpha-carbon RMSD, Supplementary Table 10).

For the other two monomers (B, C), one of the beta-alpha loops shifts to make contact with an adjacent monomer in the crystal (Supplementary Fig. 19B-C). For monomers C/B one loop undergoes a slight conformational deviation from the model, which looks like it is driven by Phe10/102 plugging into the hydrophobic barrel of the neighboring monomer. As F15N is monomeric when clean post ion exchange fractions are run on SEC, we believe F15C is very likely monomeric, and that the cross-chain interaction seen in the crystal is driven by high local concentration and consistent with crystal packing.

(Supplementary Fig 16). There might be conformational flexibility in this loop; however, all other beta-alpha loops in the symmetric structure adhere quite well to the model. This loop conformational flexibility might help maintain the stability of the barrel and could possibly help functionalize the barrel in the future.

Apart from one loop on 2 of 4 monomers, the rest of the structures adhere very well to the backbone template, and overall, the structure folds as expected.

References

1. Torng, W. & Altman, R. B. 3d deep convolutional neural networks for amino acid environment similarity analysis. BMC bioinformatics **18**, 302 (2017).

2. Shroff, R. et al. Discovery of novel gain-of-function mutations guided by structure-based deep learning. ACS Synthetic Biology **9**, 2927–2935 (2020).

3. Boomsma, W. & Frellsen, J. Spherical convolutions and their application in molecular modelling. In Advances in Neural Information Processing Systems, 3433–3443 (2017).

4. Weiler, M., Geiger, M., Welling, M., Boomsma, W. & Cohen, T. 3d steerable cnns: Learning rotationally equivariant features in volumetric data. In Advances in Neural Information Processing Systems, 10381–10392 (2018).

5. Li, B., Yang, Y. T., Capra, J. A. & Gerstein, M. B. Predicting changes in protein thermodynamic stability upon point mutation with deep 3d convolutional neural networks. PLoS computational biology **16**, e1008291 (2020).

6. Du, Y. D., Meier, J., Ma, J., Fergus, R. & Rives, A. Energy-based models for atomic-resolution protein conformations. International Conference on Learning Representations 2020 (2020).

7. Liu, K. et al. Prediction of amino acid side chain conformation using a deep neural network. arXiv preprint arXiv:1707.08381 (2017).

8. Baldwin, R. L. Energetics of protein folding. Journal of molecular biology **371**, 283–301 (2007).

9. Dill, K. A. Additivity principles in biochemistry. Journal of Biological Chemistry **272**, 701–704 (1997).

10. Kellis Jr, J. T., Nyberg, K. & Fersht, A. R. Energetics of complementary side chain packing in a protein hydrophobic core. Biochemistry **28**, 4914–4922 (1989).

11. Eriksson, A. E. et al. Response of a protein structure to cavity-creating mutations and its relation to the hydrophobic effect. Science **255**, 178–183 (1992).

12. Eriksson, A., Baase, W., Wozniak, J. & Matthews, B. A cavity-containing mutant of t4 lysozyme is stabilized by buried benzene. Nature **355**, 371 (1992).

13. Kauzmann, W. Some factors in the interpretation of protein denaturation. In Advances in protein chemistry, vol. 14, 1–63 (Elsevier, 1959).

14. Eisenberg, D. & McLachlan, A. D. Solvation energy in protein folding and binding. Nature **319**, 199 (1986).

15. Le Grand, S. M. & Merz Jr, K. M. Rapid approximation to molecular surface area via the use of boolean logic and look-up tables. Journal of Computational Chemistry **14**, 349–352 (1993).

16. Fleming, P. J. & Rose, G. D. Do all backbone polar groups in proteins form hydrogen bonds? Protein Science **14**, 1911–1917 (2005).

17. Boyken, S. E. et al. De novo design of protein homo-oligomers with modular hydrogen-bond network–mediated specificity. Science **352**, 680–687 (2016).

18. Leaver-Fay, A. et al. Chapter nineteen - rosetta3: An object-oriented software suite for the simulation and design of macromolecules. In Johnson, M. L. & Brand, L. (eds.) Computer Methods, Part C, vol. 487 of Methods in Enzymology, 545 – 574 (Academic Press, 2011). URL `http://www.sciencedirect.com/science/article/pii/B9780123812704000196`.

19. Yang, J. et al. Improved protein structure prediction using predicted interresidue orientations. Proceedings of the National Academy of Sciences **117**, 1496–1503 (2020).

20. Buchan, D. W. & Jones, D. T. The psipred protein analysis workbench: 20 years on. Nucleic acids research **47**, W402–W407 (2019).

21. Jones, D. T. Protein secondary structure prediction based on position-specific scoring matrices. Journal of molecular biology **292**, 195–202 (1999).

22. Altschul, S. F. et al. Protein database searches using compositionally adjusted substitution matrices. The FEBS journal **272**, 5101–5109 (2005).

23. Schäffer, A. A. et al. Improving the accuracy of psi-blast protein database searches with composition-based statistics and other refinements. Nucleic acids research **29**, 2994–3005 (2001).

24. Altschul, S. F. et al. Gapped blast and psi-blast: a new generation of protein database search programs. Nucleic acids research **25**, 3389–3402 (1997).

25. Kabsch, W. & Sander, C. Dictionary of protein secondary structure: pattern recognition of hydrogen-bonded and geometrical features. Biopolymers: Original Research on Biomolecules **22**, 2577–2637 (1983).

26. Madeira, F. et al. The embl-ebi search and sequence analysis tools apis in 2019. Nucleic acids research **47**, W636–W641 (2019).

27. Sheffler, W. & Baker, D. Rosettaholes: rapid assessment of protein core packing for structure prediction, refinement, design, and validation. Protein Science **18**, 229–239 (2009).

28. Nivón, L. G., Moretti, R. & Baker, D. A pareto-optimal refinement method for protein design scaffolds. PloS one **8**, e59004 (2013).

29. Conway, P., Tyka, M. D., DiMaio, F., Konerding, D. E. & Baker, D. Relaxation of backbone bond geometry improves protein energy landscape modeling. Protein Science **23**, 47–55 (2014).

30. Khatib, F. et al. Algorithm discovery by protein folding game players. Proceedings of the National Academy of Sciences **108**, 18949–18953 (2011).

31. Tyka, M. D. et al. Alternate states of proteins revealed by detailed energy landscape mapping. Journal of molecular biology **405**, 607–618 (2011).

32. Huang, P.-S. et al. De novo design of a four-fold symmetric tim-barrel protein with atomic-level accuracy. Nature chemical biology **12**, 29 (2016).

33. Caldwell, S. J. et al. Tight and specific lanthanide binding in a de novo tim barrel with a large internal cavity designed by symmetric domain fusion. Proceedings of the National Academy of Sciences **117**, 30362–30369 (2020).

34. Romero-Romero, S. et al. The stability landscape of de novo tim barrels explored by a modular design approach. Journal of molecular biology **433**, 167153 (2021).

Supplementary Figures

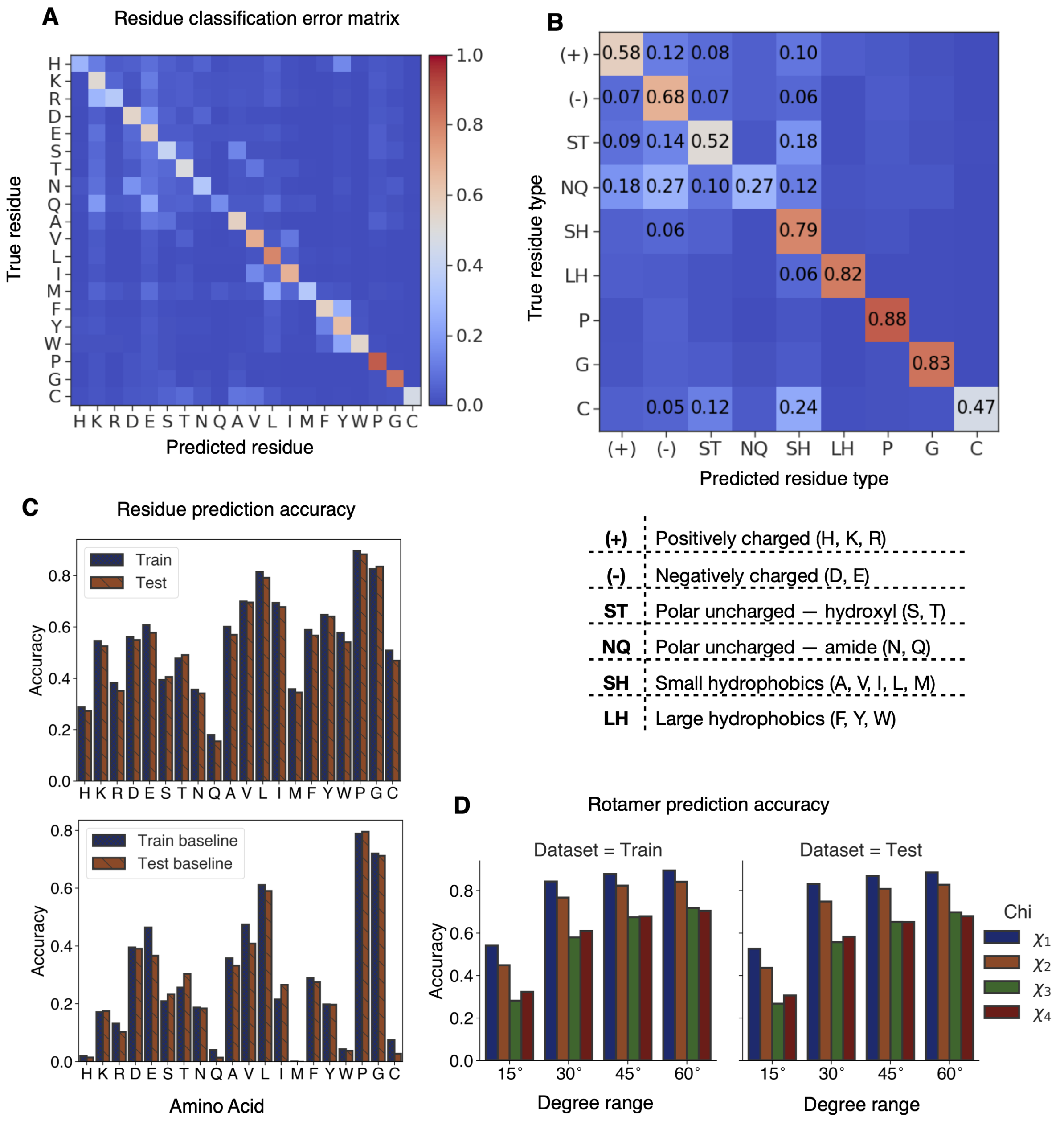

Supplementary Figure 1: Classifier performance. **(A)** Classifier error matrix for individual residue prediction on test set data and for **(B)** prediction of groups of biochemically similar residues. **(C)** Residue-specific prediction accuracy for train and test set data for the (top) conditional model and (bottom) baseline model. **(D)** Rotamer prediction accuracy to within 15, 30, 45, and 60 degrees across the train and test set.

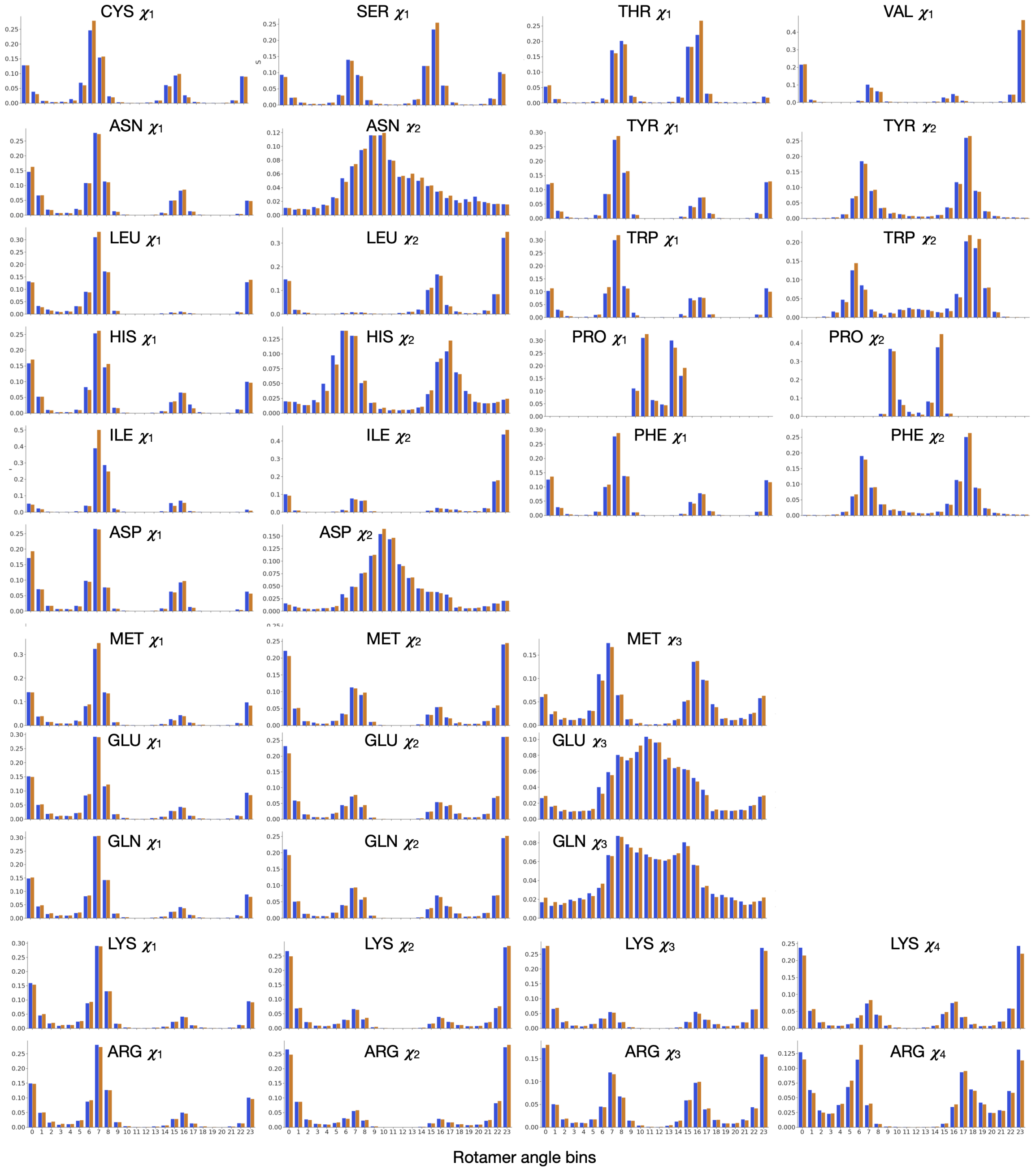

Supplementary Figure 2: Learned residue-specific rotamer distributions. Binned rotamer distributions for the native test set domains (blue) versus the model predicted distributions (orange). Native distributions are the normalized empirical rotamer distributions for each residue across the test set. Model distributions are the average of the network-predicted distributions across the test set examples.

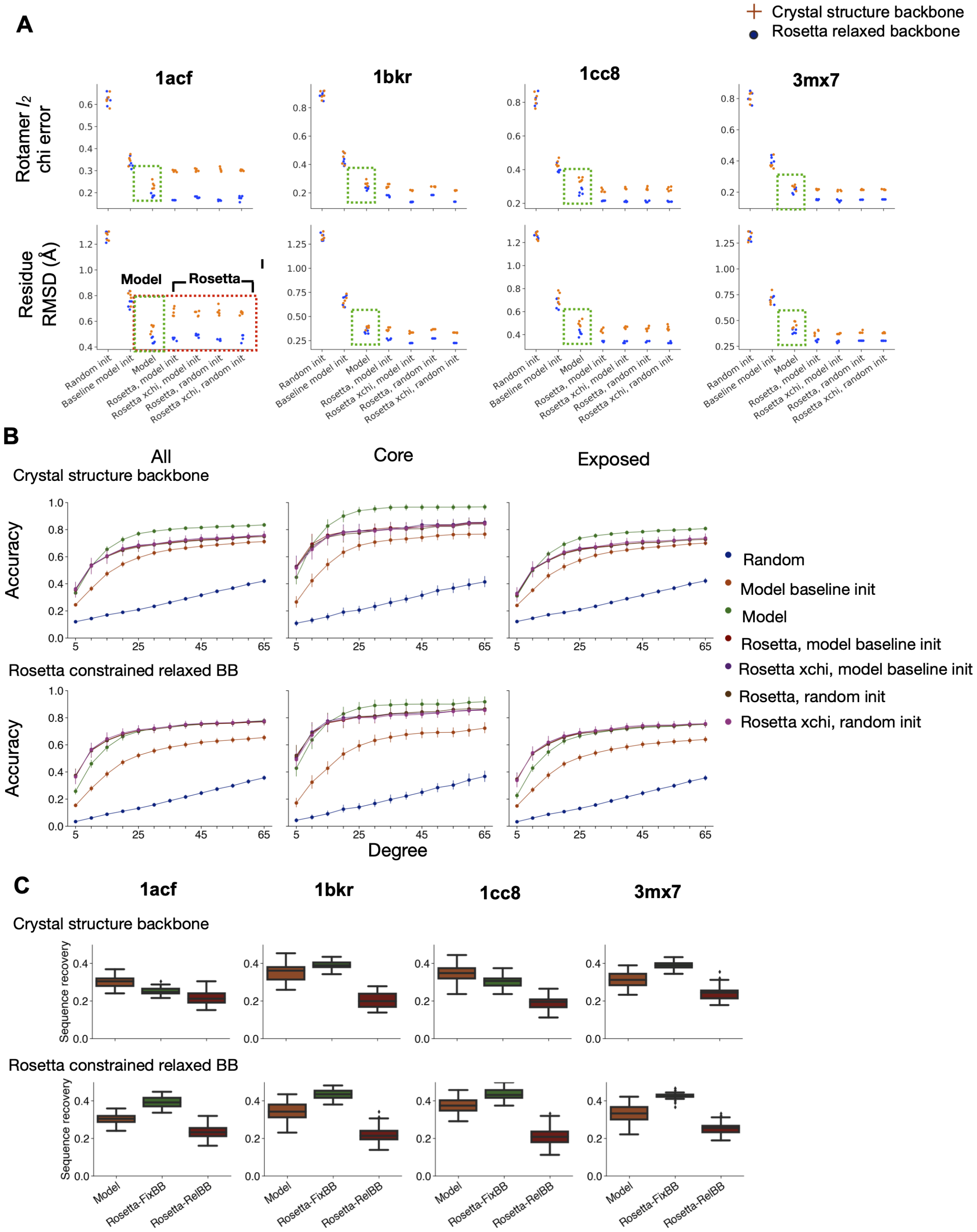
A
Crystal structure backbone
Rosetta relaxed backbone
1acf
1bkr
1cc8
3mx7
Rotamer l_2 chi error
Residue RMSD (Å)
Model
Rosetta
Random init
Baseline model init
Model
Rosetta, model init
Rosetta xchi, model init
Rosetta, random init
Rosetta xchi, random init
B
All
Core
Exposed
Crystal structure backbone
Rosetta constrained relaxed BB
Accuracy
Degree
Random
Model baseline init
Model
Rosetta, model baseline init
Rosetta xchi, model baseline init
Rosetta, random init
Rosetta xchi, random init
C
1acf
1bkr
1cc8
3mx7
Crystal structure backbone
Rosetta constrained relaxed BB
Sequence recovery
Model
Rosetta-FixBB
Rosetta-RelBB

Supplementary Figure 3 *(preceding page)*: Rotamer and native sequence recovery. (**A - B**) Rotamer recovery. $n = 5$ for all methods for each test case. Data shown for rotamer recovery on crystal structure backbone, as well as Rosetta constrained relaxed backbones. Random init – random initialization of chi angles. Baseline model init – initialization with rotamer prediction by baseline classifier model from backbone alone. Model – rotamer sampling and annealing with conditional model. Rosetta protocols were run either with or without extra χ^1 and χ^2 sampling (xchi). **(A)** (Top) Average l_2 chi angle error across design methods. (Bottom) Average residue side-chain RMSD (Å). **(B)** Rotamer recovery accuracy as a function of degree cutoff for all residues, core residues, and solvent-exposed residues ($n = 5$, each). Data are presented as mean values with 95% CI. **(C)** Native sequence recovery across test case crystal structures ($n = 50$, each). (Top) Design on crystal structures. (Bottom) Design on Rosetta constrained relaxed backbones. Box plots rendered with median center, bounds of boxes corresponding to interquartile range (IQR), whisker length 1.5*IQR, and outliers outside of 1.5*IQR range.

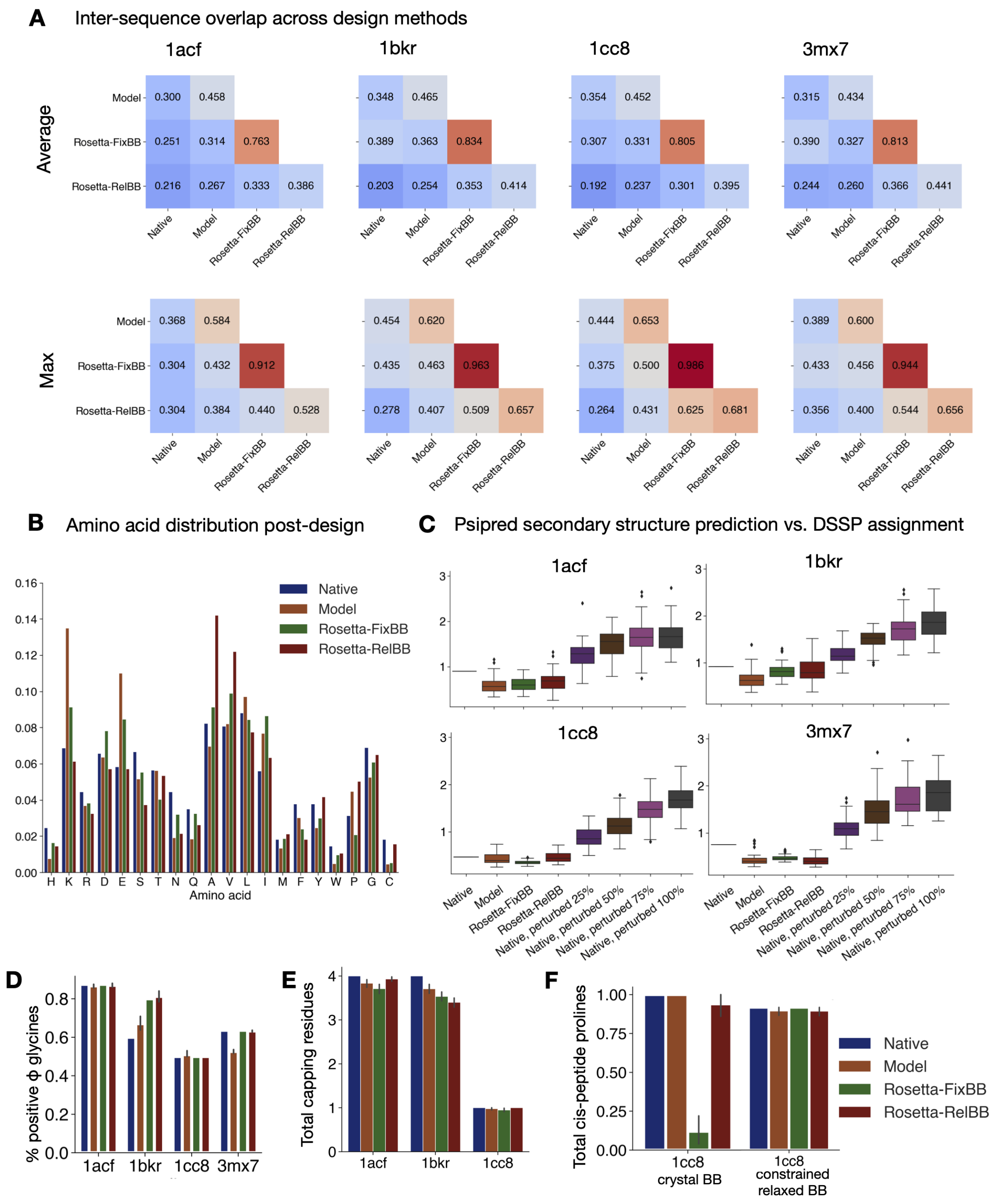
A
Inter-sequence overlap across design methods
1acf
1bkr
1cc8
3mx7
Average
Max
Model
Rosetta-FixBB
Rosetta-RelBB
Native
0.300 0.458
0.251 0.314 0.763
0.216 0.267 0.333 0.386
0.348 0.465
0.389 0.363 0.834
0.203 0.254 0.353 0.414
0.354 0.452
0.307 0.331 0.805
0.192 0.237 0.301 0.395
0.315 0.434
0.390 0.327 0.813
0.244 0.260 0.366 0.441
0.368 0.584
0.304 0.432 0.912
0.304 0.384 0.440 0.528
0.454 0.620
0.435 0.463 0.963
0.278 0.407 0.509 0.657
0.444 0.653
0.375 0.500 0.986
0.264 0.431 0.625 0.681
0.389 0.600
0.433 0.456 0.944
0.356 0.400 0.544 0.656
B
Amino acid distribution post-design
Amino acid
H K R D E S T N Q A V L I M F Y W P G C
C
Psipred secondary structure prediction vs. DSSP assignment
Native, perturbed 25%
Native, perturbed 50%
Native, perturbed 75%
Native, perturbed 100%
D
% positive ϕ glycines
E
Total capping residues
F
Total cis-peptide prolines
1cc8 crystal BB
1cc8 constrained relaxed BB

Supplementary Figure 4 ***(preceding page)*****:** Assessing model designs in comparison to Rosetta baselines. **(A)** Inter-sequence percent overlap. Average (top) and maximum (bottom) sequence percent identity within and across top sequences from each design method. **(B)** Amino acid distribution post-design relative to native sequence and aligned homologs (max. 500 hits per sequence). MSAs obtained using PSI-BLAST v2.9, the NR50 database, BLOSUM62 with an E-value cutoff of 0.1. **(C)** Psipred secondary structure prediction for designed sequences. Cross-entropy of Psipred prediction (helix, loop, sheet) from sequence alone with respect to DSSP assignments (*20–25*). Box plots rendered with median center, bounds of boxes corresponding to interquartile range (IQR), whisker length 1.5*IQR, and outliers outside of 1.5*IQR range. **(D-F)** Designed structural features. Data are presented as mean values with 95% CI. **(D)** Percent glycines at positive ϕ backbone positions across test cases ($n = 50$, each). **(E)** Capping residue placement. Average number of N-terminal helical capping residues across designs for test cases with capping positions ($n = 50$, each). **(F)** Total number of cis-peptide prolines ($|\omega_{i-1}| < 15$) for *1cc8* for designs on crystal structure backbone vs. Rosetta constrained relaxed backbone ($n = 50$).

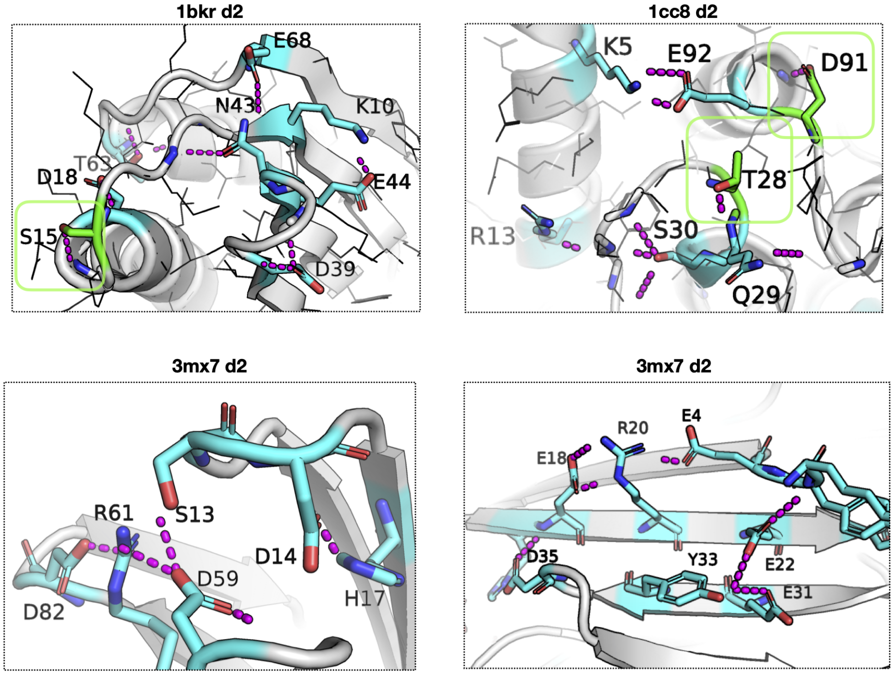

Supplementary Figure 5: Examples of polar networks designed by the model on native test cases. N-terminal helical capping residues highlighted in green.

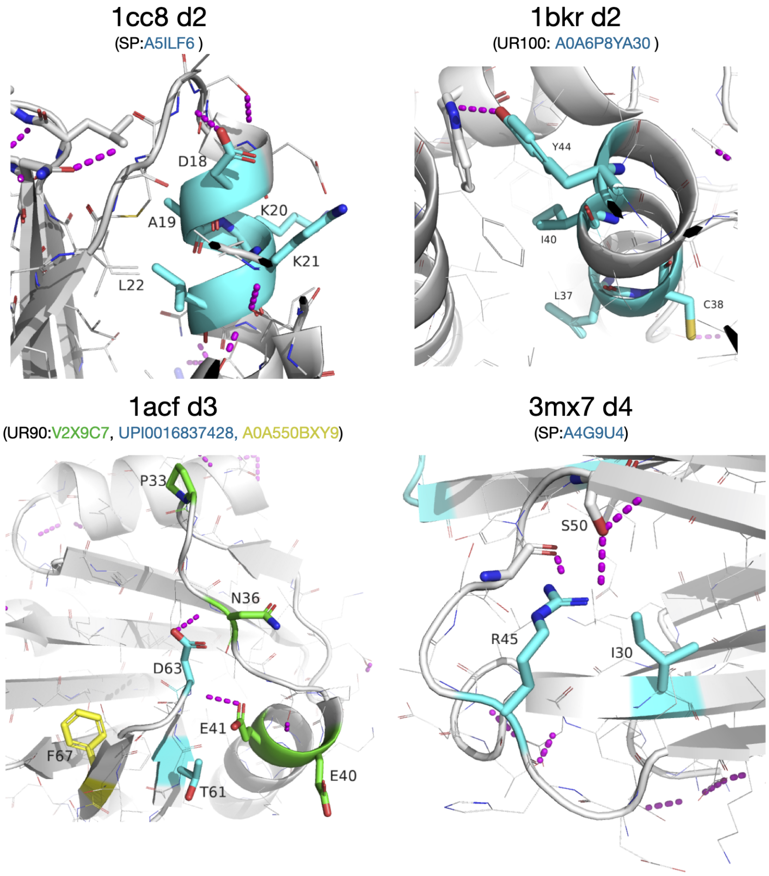

Supplementary Figure 6: Examples of model design features not seen in native sequence, yet seen in homologous sequences found via multiple sequence alignment again sequence databases (UniRef90 (UR90), UniRef100 (UR100), UniProtKB/Swiss-Prot (SP)) (*26*). Homologous sequence accession codes given above each panel. Colored residues are residues from model design found in homologous sequence and not in native sequence.

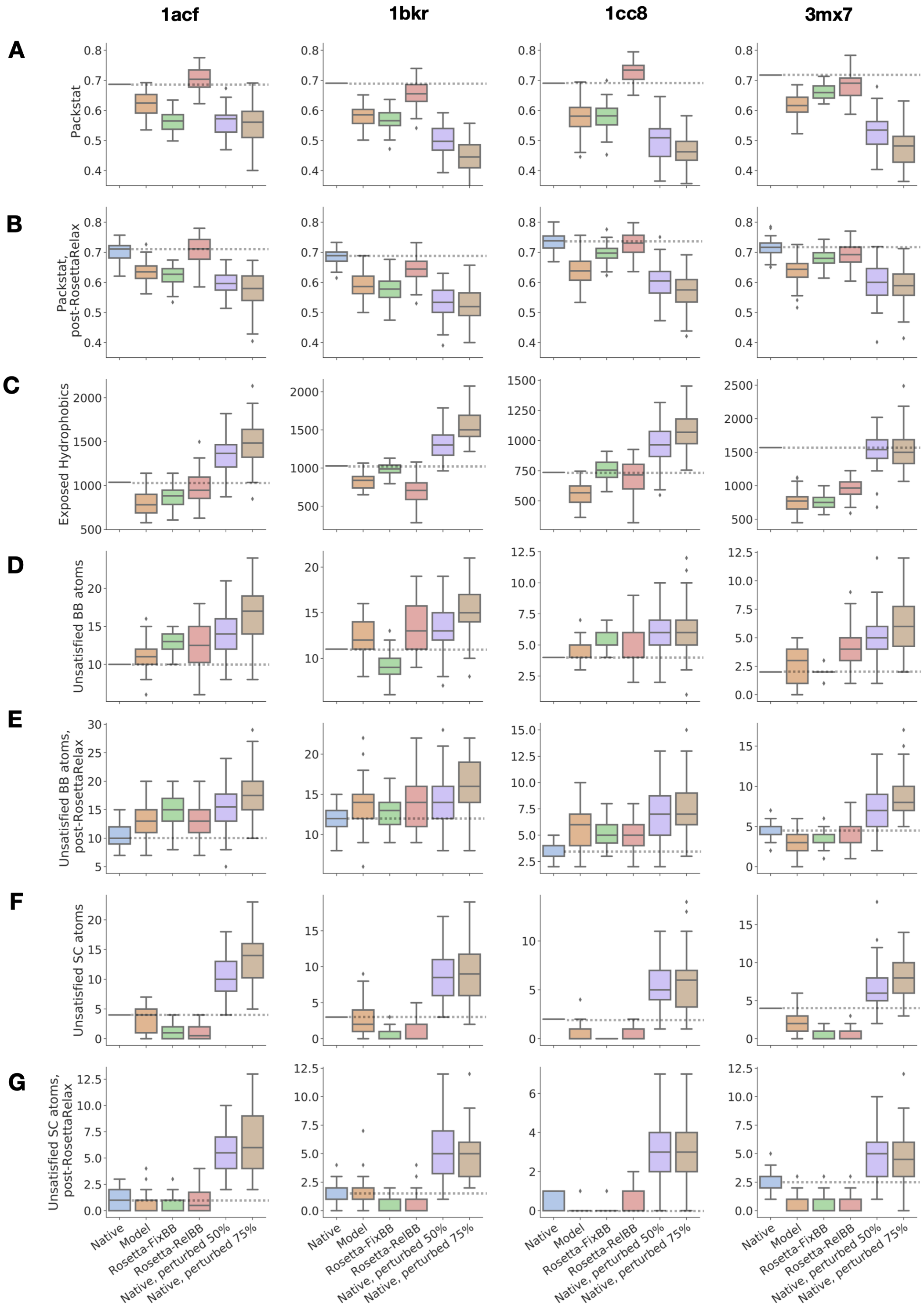
1acf
1bkr
1cc8
3mx7
A
B
C
D
E
F
G
Packstat
Packstat, post-RosettaRelax
Exposed Hydrophobics
Unsatisfied BB atoms
Unsatisfied BB atoms, post-RosettaRelax
Unsatisfied SC atoms
Unsatisfied SC atoms, post-RosettaRelax
Native
Model
Rosetta-FixBB
Rosetta-RelBB
Native, perturbed 50%
Native, perturbed 75%

Supplementary Figure 7 ***(preceding page)*****:** Biochemical metrics of interest for designs on crystal structures. Box plots rendered with median center, bounds of boxes corresponding to interquartile range (IQR), whisker length 1.5*IQR, and outliers outside of 1.5*IQR range. Designs ($n = 50$) compared to native crystal structure ($n = 1$) or distribution of relaxed native structures ($n = 50$). 50% and 75% mutated native sequences included as negative controls. (**A-B**) Packstat, a measure of core residue packing (*27*) pre- **(A)** and post- **(B)** RosettaRelax (*28–31*). **(C)** Total solvent-accessible surface area (SASA) of exposed hydrophobic residues (*15*). (**D-E**) Number of buried unsatisfied polar backbone (BB) atoms pre- **(D)** and post- **(E)** relax. (**F-G**) Number of buried unsatisfied polar side-chain (SC) atoms pre- **(F)** and post- **(G)** relax.

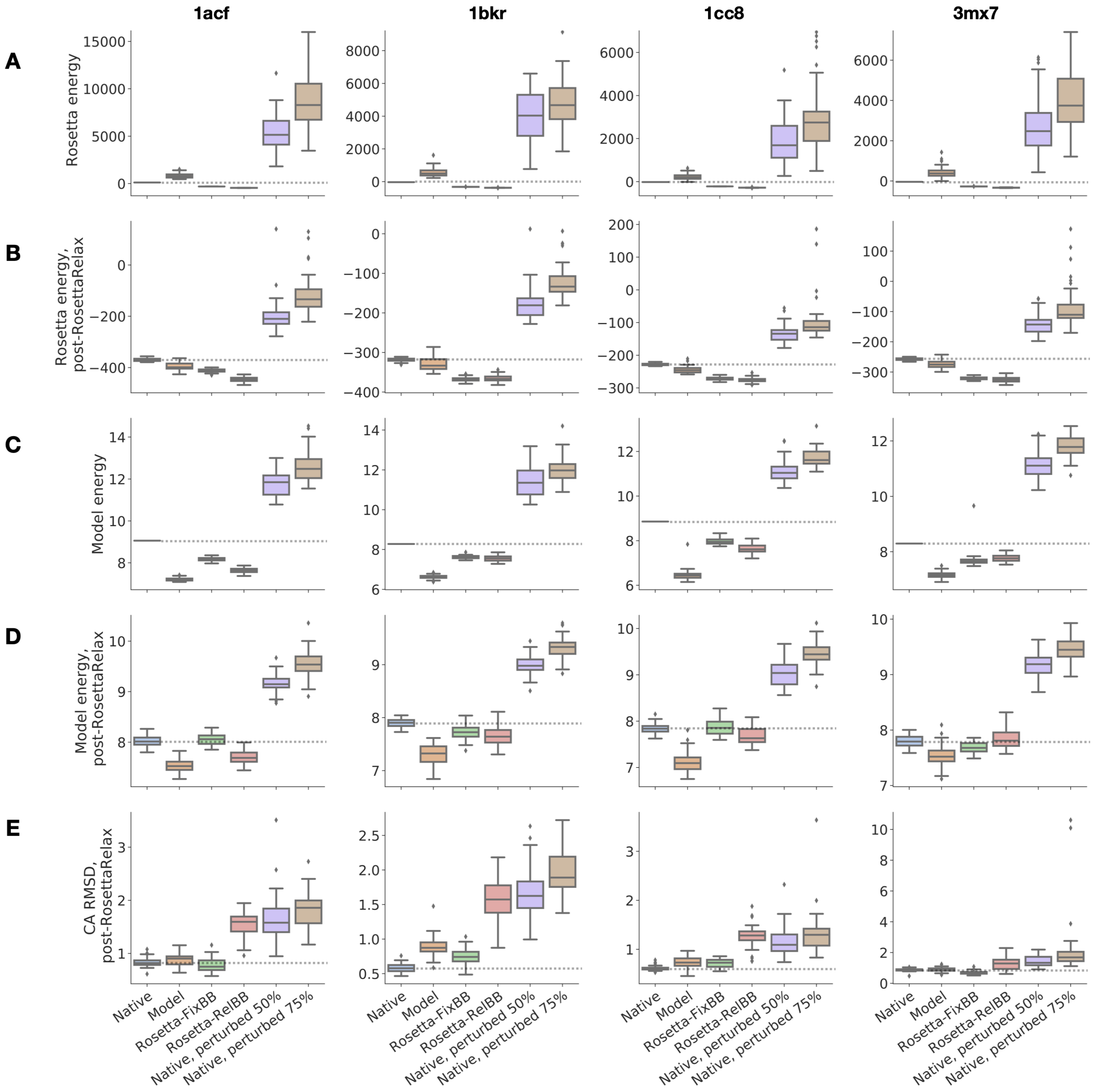

Supplementary Figure 8: Energy under the model and under the Rosetta energy function pre- and post-RosettaRelax. 50% and 75% mutated native sequences included as negative controls. Designs ($n = 50$) compared to native crystal structure ($n = 1$) or distribution of relaxed native structures ($n = 50$). Box plots rendered with median center, bounds of boxes corresponding to interquartile range (IQR), whisker length 1.5*IQR, and outliers outside of 1.5*IQR range. (**A-B**) Rosetta energy pre- **(A)** and post- **(B)** RosettaRelax. (**C-D**) Model energy (negative pseudo-log-likelihood, normalized by protein length) pre- **(C)** and post- **(D)** relax. **(E)** Alpha-carbon RMSD (Å) post-RosettaRelax.

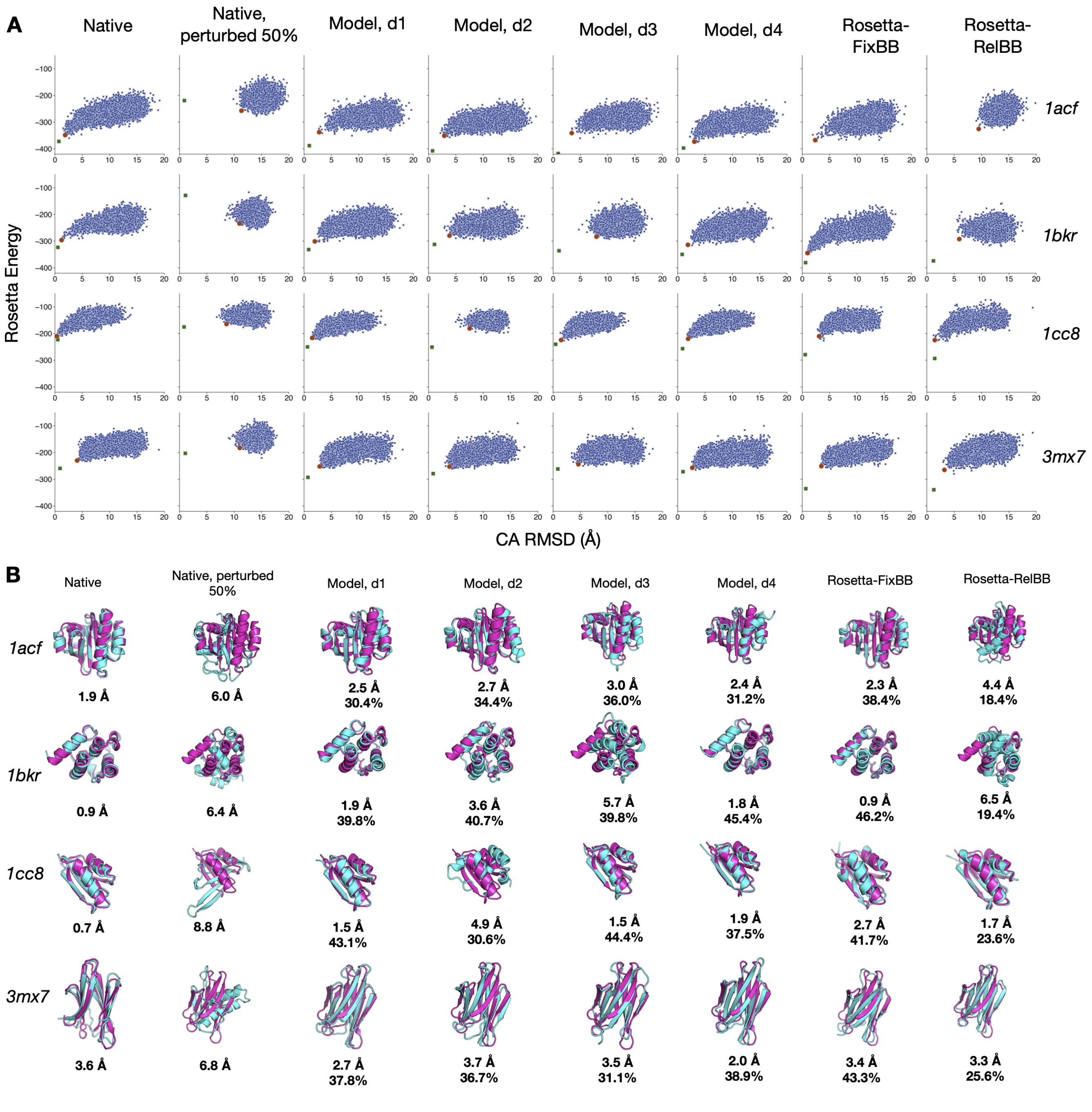

Supplementary Figure 9: Rosetta AbInitio structure prediction of designed native test case sequences and Rosetta-FixBB and Rosetta-RelBB baselines. 50% randomly perturbed native sequences included as a negative control. Rosetta designs and perturbed sequences are the best of 50 sequences by Rosetta energy. **(A)** Rosetta energy vs. RMSD (Å) to native funnel plots. Selected structure with best summed rank of template-RMSD and Rosetta energy is shown in orange. The designed sequence after RosettaRelax is shown in green. **(B)** Folded structure with best summed rank of template-RMSD and Rosetta energy across 10^4 folding trajectories. Decoys (blue) are aligned to the native backbones (pink). Sequence identity and RMSD (Å) compared to native are reported below the structures.

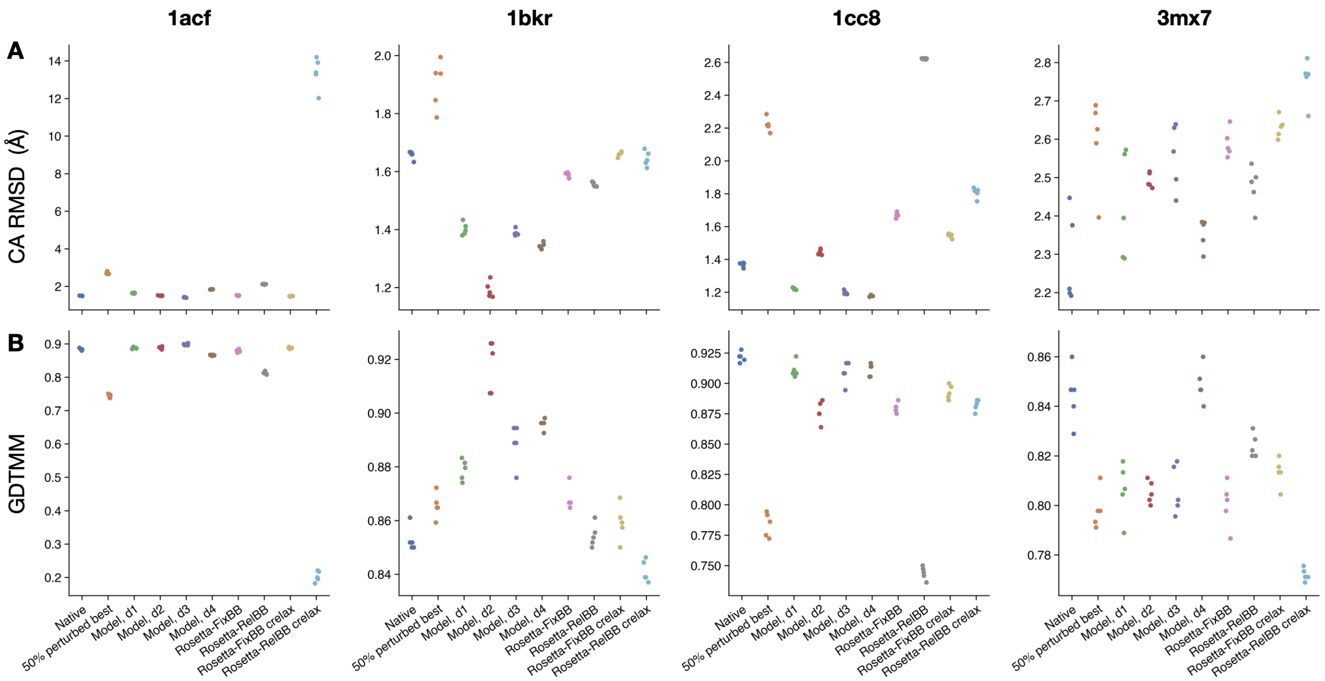

Supplementary Figure 10: trRosetta structure prediction of designed native test case sequences and baselines. Data for 5 predicted 3D structure models. Data shown for native sequence, 50% perturbed native sequence (best of 50 sequences in Rosetta energy), model designs, best of 50 Rosetta-FixBB and Rosetta-RelBB designs on crystal structure (in terms of Rosetta energy), and best of 50 Rosetta-FixBB and Rosetta-RelBB designs on the constrained relaxed crystal backbone. **(A)** Alpha-carbon (CA) RMSD (Å) to native. **(B)** GDTMM score (higher is better).

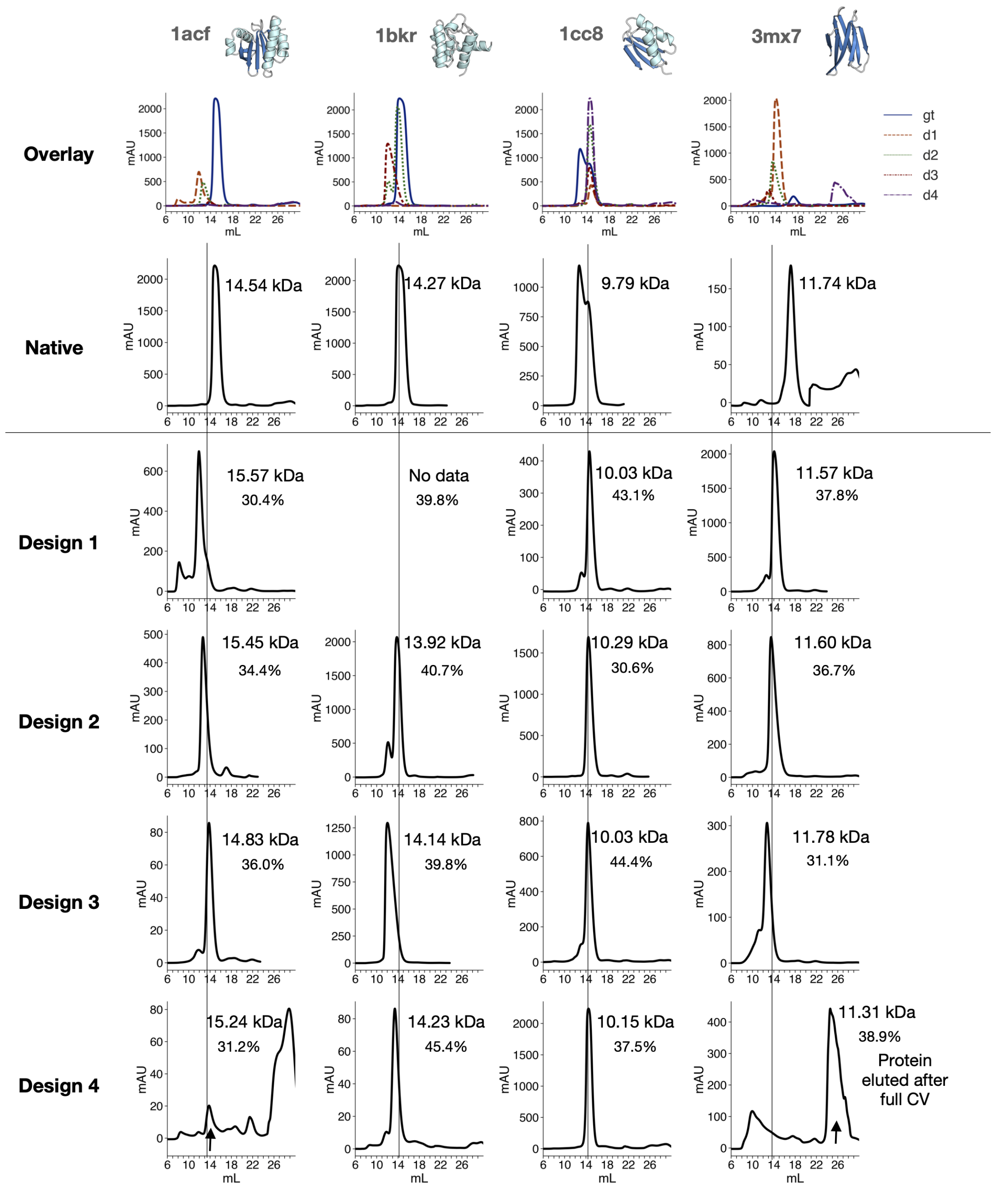

Supplementary Figure 11: Determining sample quality via size-exclusion chromatography (Superdex 75) done immediately post-NiNTA purification without additional purification steps. Sequence identity to native reported below construct name. Theoretical molecular weight based on the sequence reported within each panel. Vertical line shows expected elution volume based on molecular weight of the native sequence. Some proteins seem to interact with the column, eluting later than expected (*3mx7* native, *3mx7* d4). No data for *1bkr* d1 due to low protein expression. Right-most peak for *1acf* d4 is imidazole.

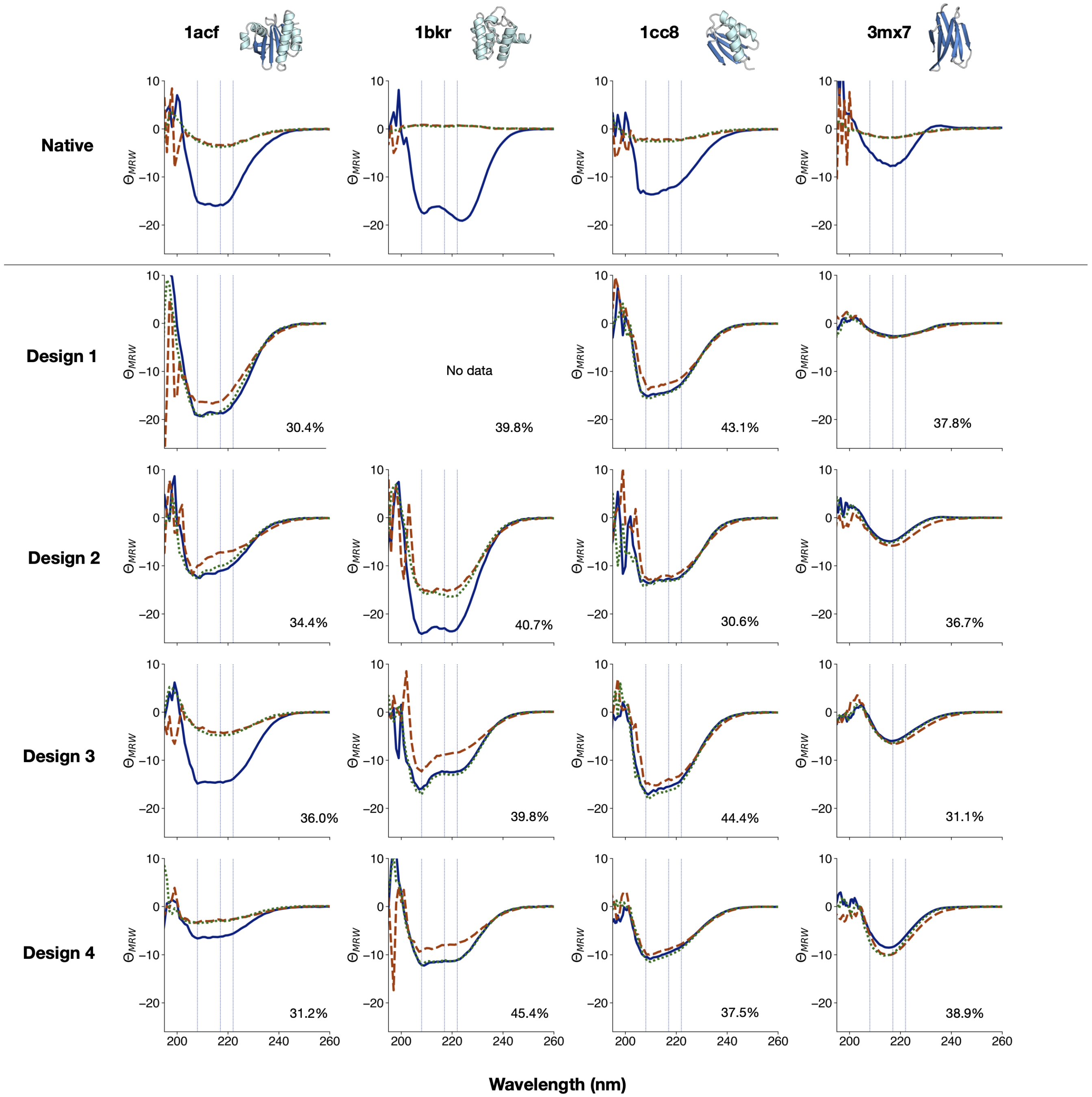

Supplementary Figure 12: Circular dichroism (CD) spectroscopy wavelength scans for select model designs on native test case backbones. Sequence identity to native reported below construct name. Mean residue ellipticity Θ_{MRW} (10^3deg cm^2 dmol^{-1}) for CD wavelength scans at 20°C (blue, solid), melted at 95°C (orange, dashed), and cooled again to 20°C (green, dashed). No data for *1bkr* d1 due to low protein expression. Top sequences highlighted in main paper in Fig. 2I-J are *1acf d3*, *1bkr d2*, *1cc8 d2*, and *3mx7 d4*.

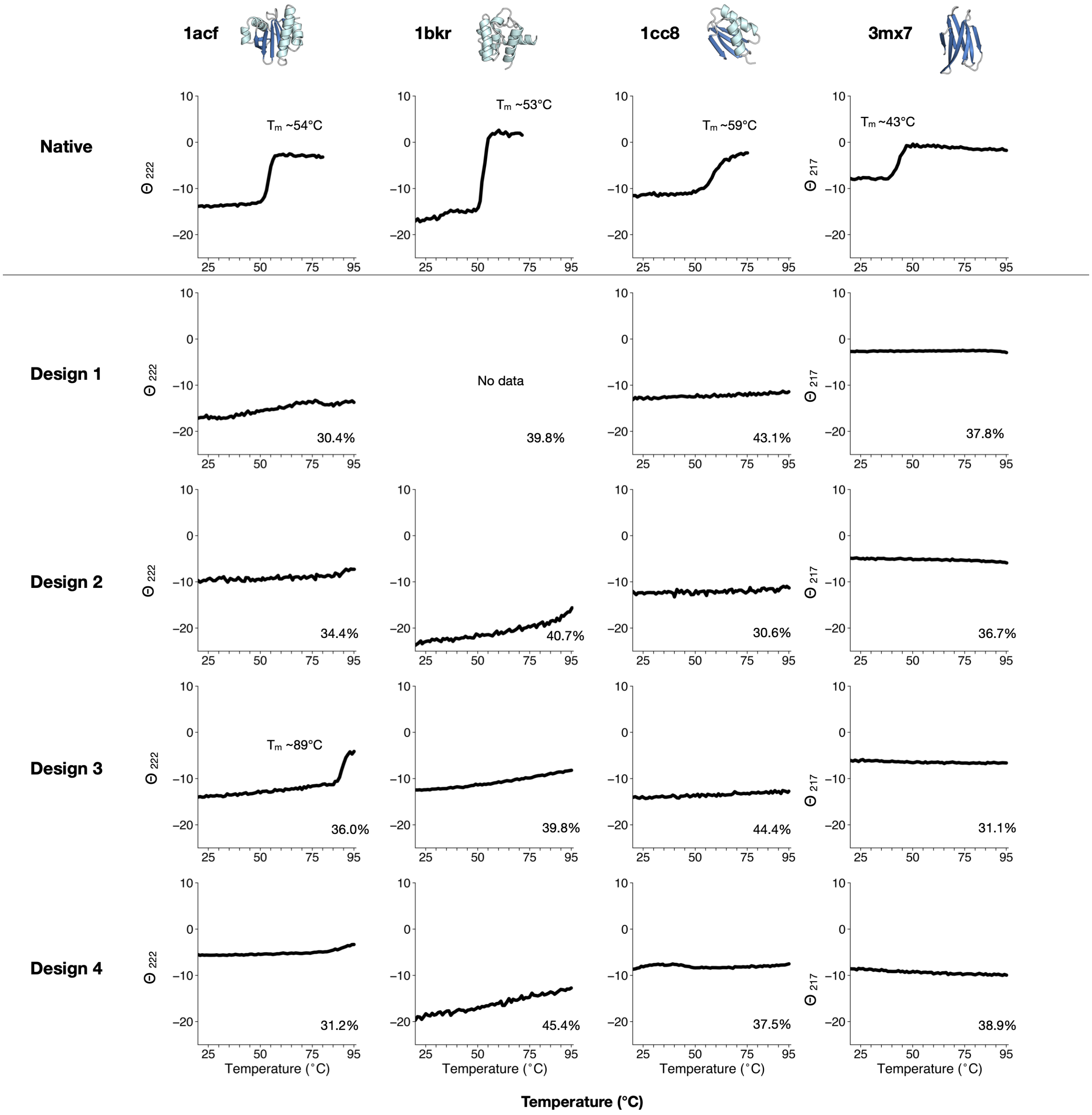

Supplementary Figure 13: Thermal melting curves for native test case designs. Sequence identity to native reported below construct name. Curves monitoring circular dichroism (CD) signal θ_{MRW} (10^3deg cm^2 dmol^{-1}) at 222nm or 217nm for *3mx7* designs. No data for *1bkr* d1 due to low protein expression. For melts with a cooperative transition, melting temperatures T_m listed. Top sequences highlighted in main paper in Fig. 2I-J are *1acf d3*, *1bkr d2*, *1cc8 d2*, and *3mx7 d4*.

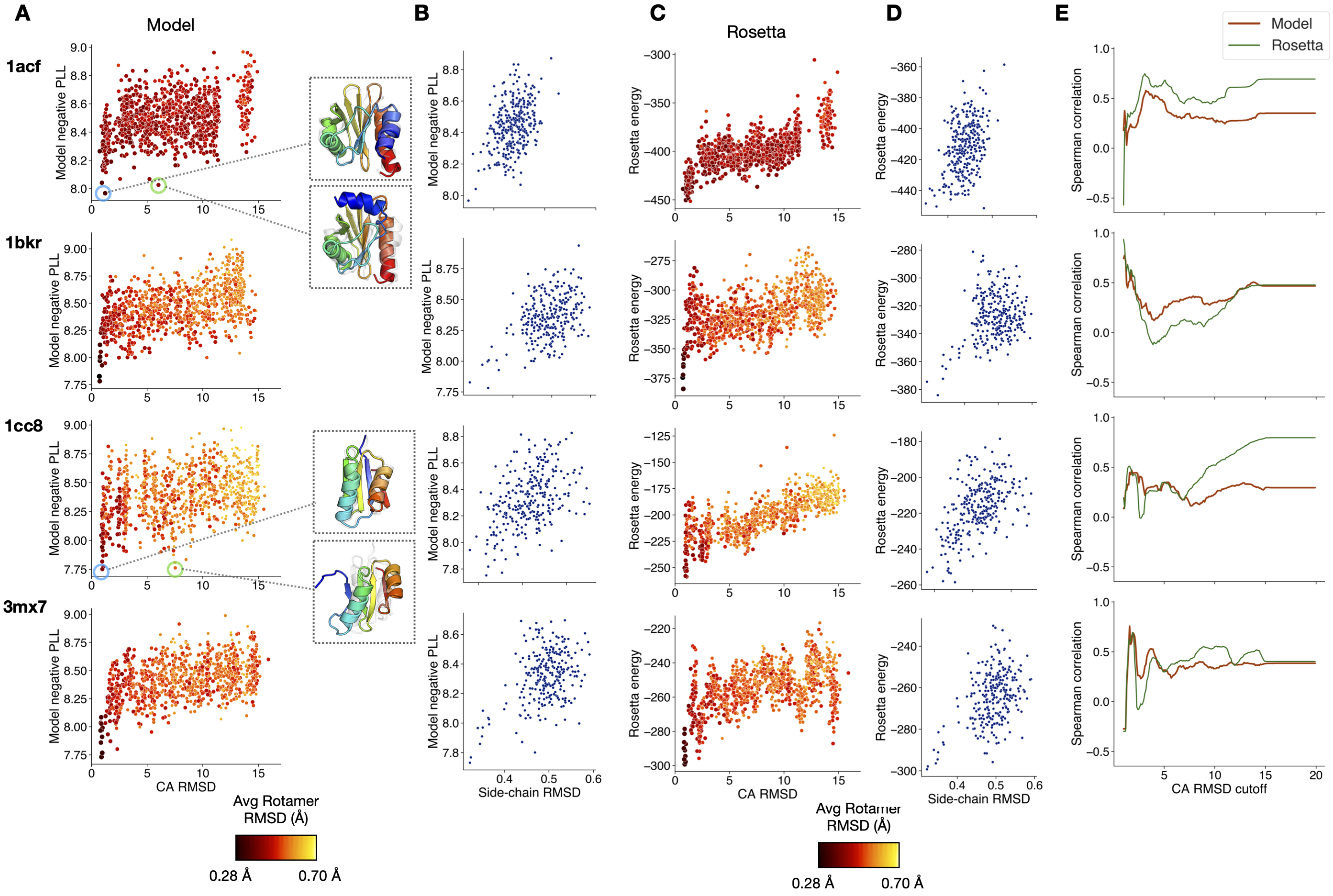

Supplementary Figure 14: Decoy ranking for test cases. **(A)** Model energy (negative PLL) vs. alpha-carbon RMSD (Å) for Rosetta AbInitio folded structures (decoys). Points are colored by average side-chain RMSD (Å) to native. (Inset) Select structures rendered to visualize alternative minima under model ranking for *1acf* and *1cc8*: for *1acf* an alternative N-terminal helix conformation and for *1cc8* an alternative pattern of beta strand pairing. **(B)** Model negative PLL of low backbone RMS structures (CA RMSD < 5 Å) vs. average side-chain RMSD (Å). **(C)** Rosetta energy vs. alpha carbon RMSD (Å) for folded structures. Points are colored by average side-chain RMSD. **(D)** Rosetta energy of low backbone RMS structures (CA RMSD < 5 Å) vs. average side-chain RMSD (Å). **(E)** Spearman rank correlation between model negative PLL/ Rosetta energy and structure alpha-carbon RMSD (Å) as a function of increasing RMSD cutoff. In the low RMS regime (< 5 Å), the model and Rosetta are able to rank low RMS structures to a similar extent.

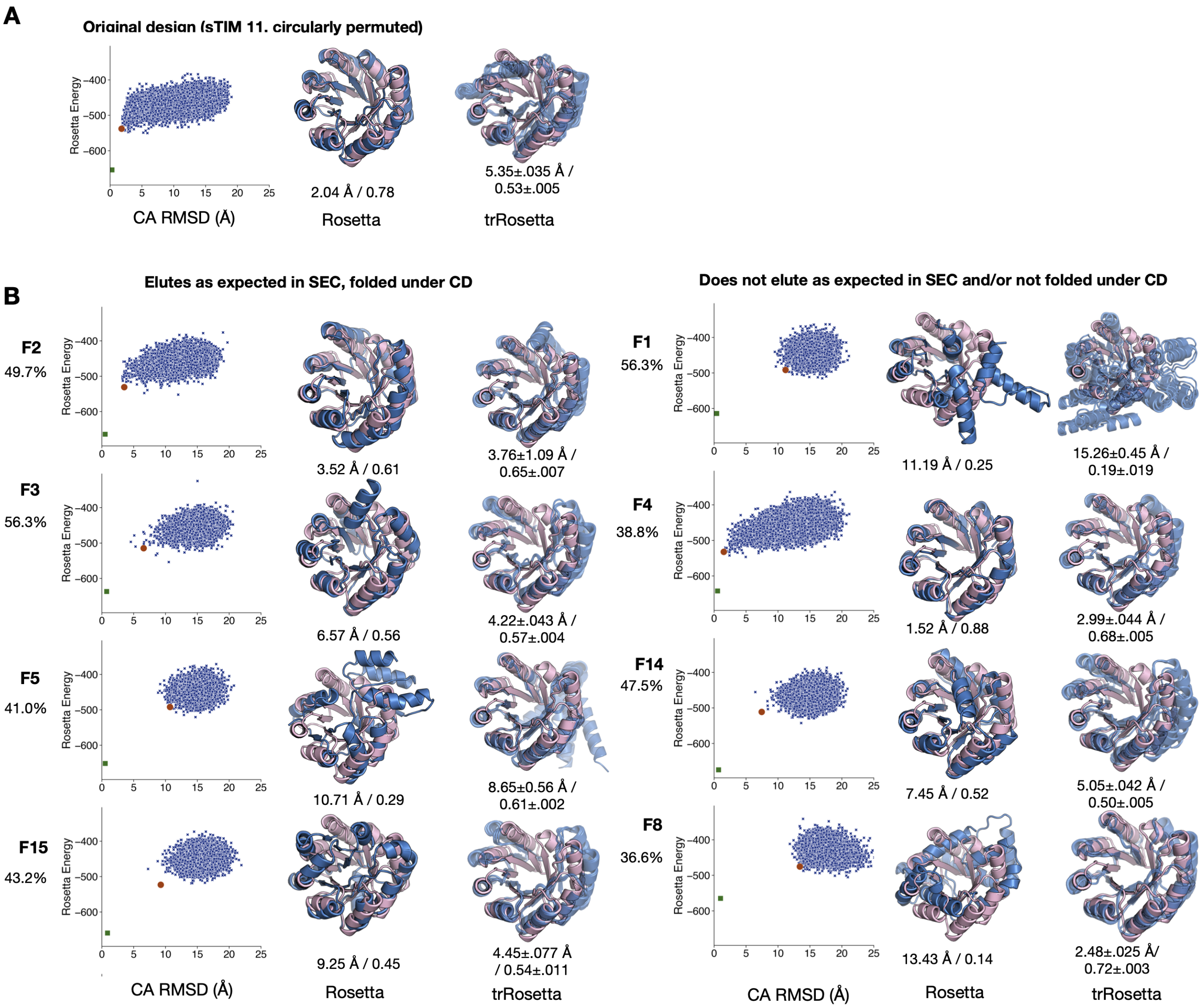

Supplementary Figure 15: Structure prediction for TIM-barrel designs. Prediction for **(A)** original sTIM-11 sequence (circularly permuted with mutations C8Q, C181V) and (**B**) TIM-barrel model designs. Sequence identity to sTIM11 reported below construct name. **(Left)** Rosetta AbInitio structure prediction Rosetta energy vs. RMSD (Å) to native funnel plots. Selected structure with best summed rank of template-RMSD and Rosetta energy is shown in orange. The design after RosettaRelax is shown in green. **(Center)** Folded structure with best summed rank of template-RMSD and Rosetta energy across 10^4 folding trajectories. Decoys (blue) are aligned to the target backbone (pink). Backbone alpha-carbon RMSD (Å) and GDTMM are reported below the structures. **(Right)** trRosetta prediction results. 5 models (blue) overlaid on target scaffold (pink). Backbone alpha-carbon RMSD (Å) and GDTMM below structures.

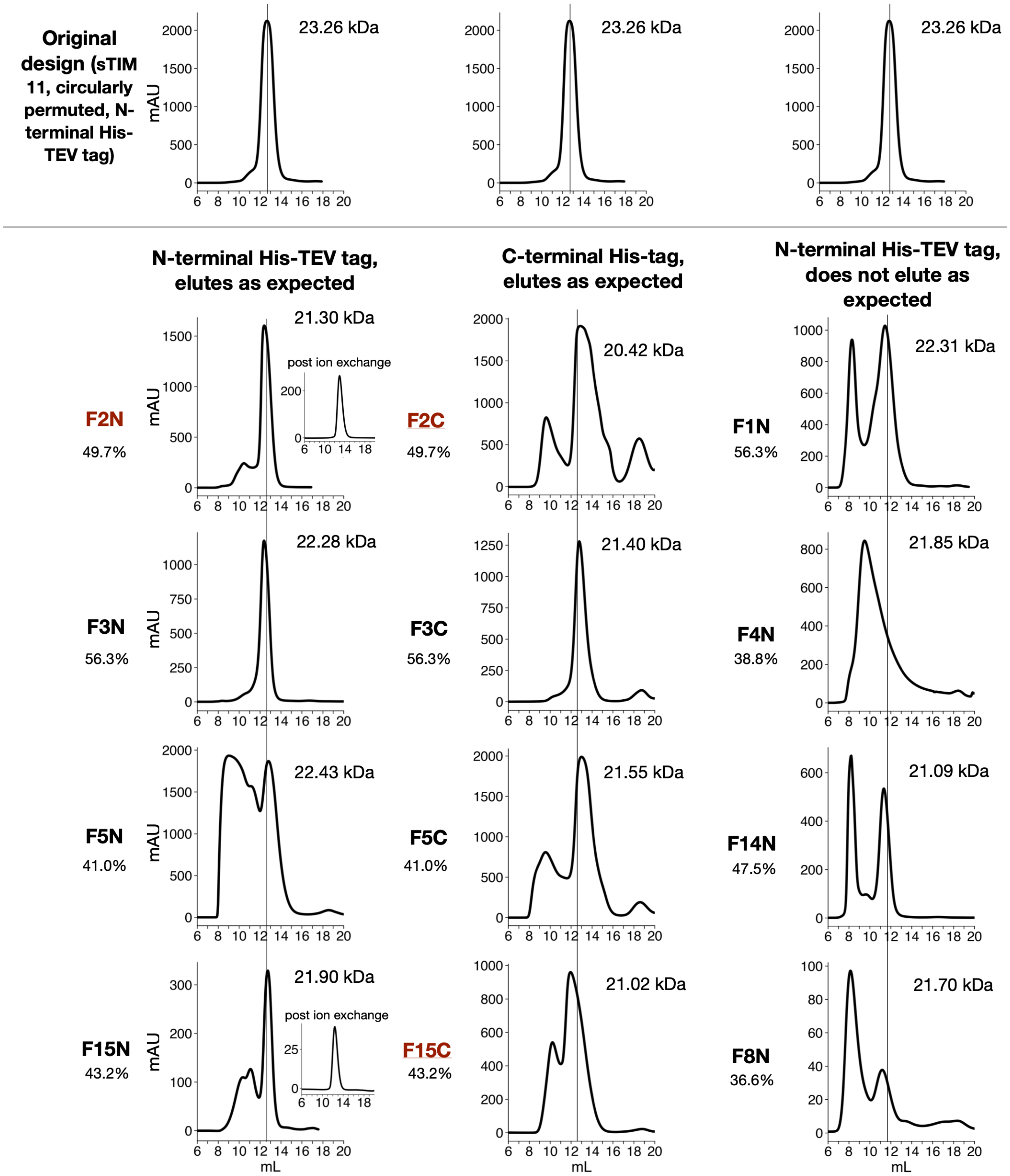

Supplementary Figure 16: Determining sample quality for model TIM-barrel designs designs via size-exclusion chromatography (Superdex 75) done immediately post-NiNTA purification without additional purification steps. Crystal structure constructs F2C and F15C highlighted in red. Sequence identity to sTIM11 reported below construct name. Theoretical molecular weight based on the sequence reported within each panel. Vertical line shows expected elution volume based on molecular weight of the sTIM-11 sequence. **(Left)** FXN N-terminal His-TEV tagged constructs that eluted as expected. **(Inset)** SEC data for post ion exchange clean fractions for F2N and F15N, showing monomeric state in solution. **(Center)** FXN C-terminal His-tagged constructs. **(Right)** FXN N-terminal His-TEV tagged constructs that did not elute as expected.

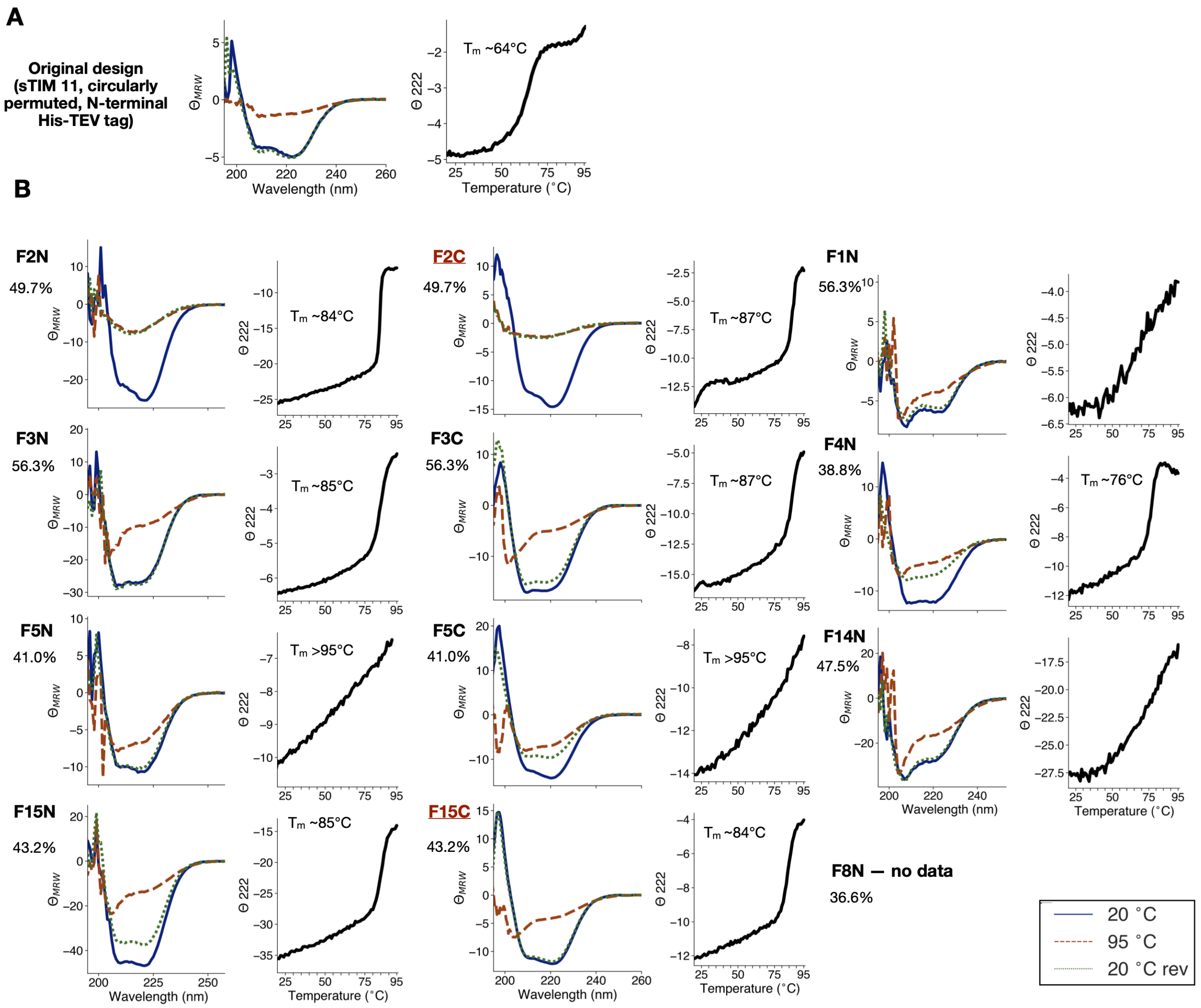

Supplementary Figure 17: Circular dichroism (CD) data for TIM-barrel structures. Sequence identity to sTIM11 reported below construct name. (**A** - **B**) Circular dichroism (CD) data for (**A**) original sTIM-11 sequence, (circularly permuted with mutations C8Q, C181V) with N-terminal His-TEV tag and (**B**) TIM-barrel model designs: FXN (N-terminal His-TEV tag), FXC (C-terminal His tag). Crystal structure constructs F2C and F15C highlighted in red. **(Left)** Mean residue ellipticity Θ_{MRW} (10^3deg cm^2 dmol^{-1}) for CD wavelength scans at 20°C (blue, solid), melted at 95°C (orange, dashed), and cooled again to 20°C (green, dashed). **(Right)** Thermal melting curves monitoring CD signal θ_{MRW} (10^3deg cm^2 dmol^{-1}) at 222nm.

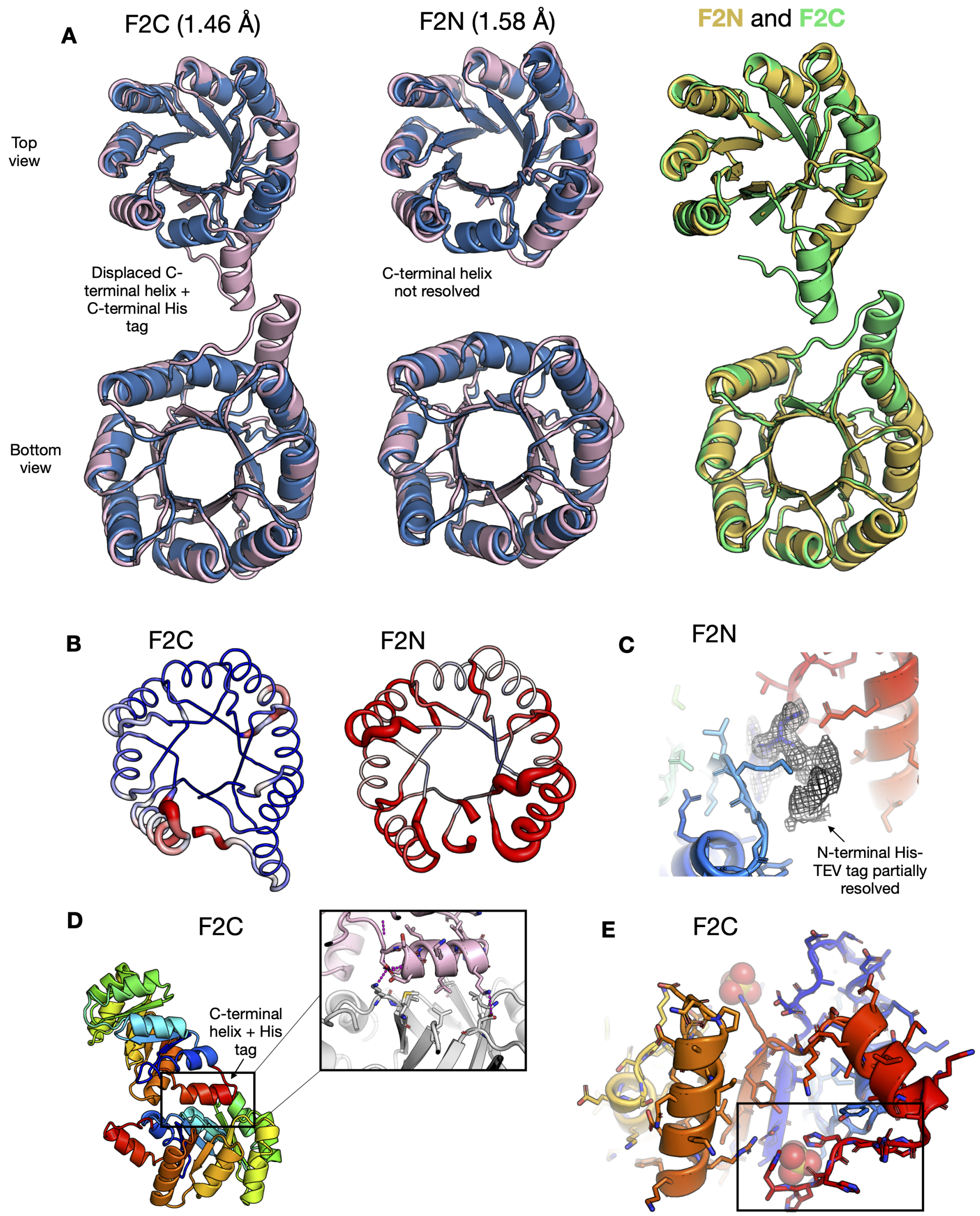

Supplementary Figure 18: Additional crystal structure data for TIM-barrel F2. **(A)** Overlay with design model (blue) for F2 with C-terminal His tag (F2C, pink, left), F2 with N-terminal His-TEV tag (F2N, pink, middle), and overlay of F2C (green) and F2N (yellow) crystal structures (right). **(B)** F2C and F2N crystal structures colored by B-factor. **(C)** Resolved density for part of the N-terminal His-TEV tag for F2N, showing the tag packing against the barrel, displacing the C-terminal short helix. **(D)** The C-terminal helix of the TIM-barrel dislodges from the barrel and makes contact with the bottom of the barrel of an adjacent protein. (Inset) The C-terminal helix plugs into hydrophobic residues in the barrel, while also making polar contacts. **(E)** Close-up of the C-terminal helix and the resolved His tag, showing displacement of the C-terminal short helix and binding of a sulfate ion which coordinates His 186 and 188 (box).

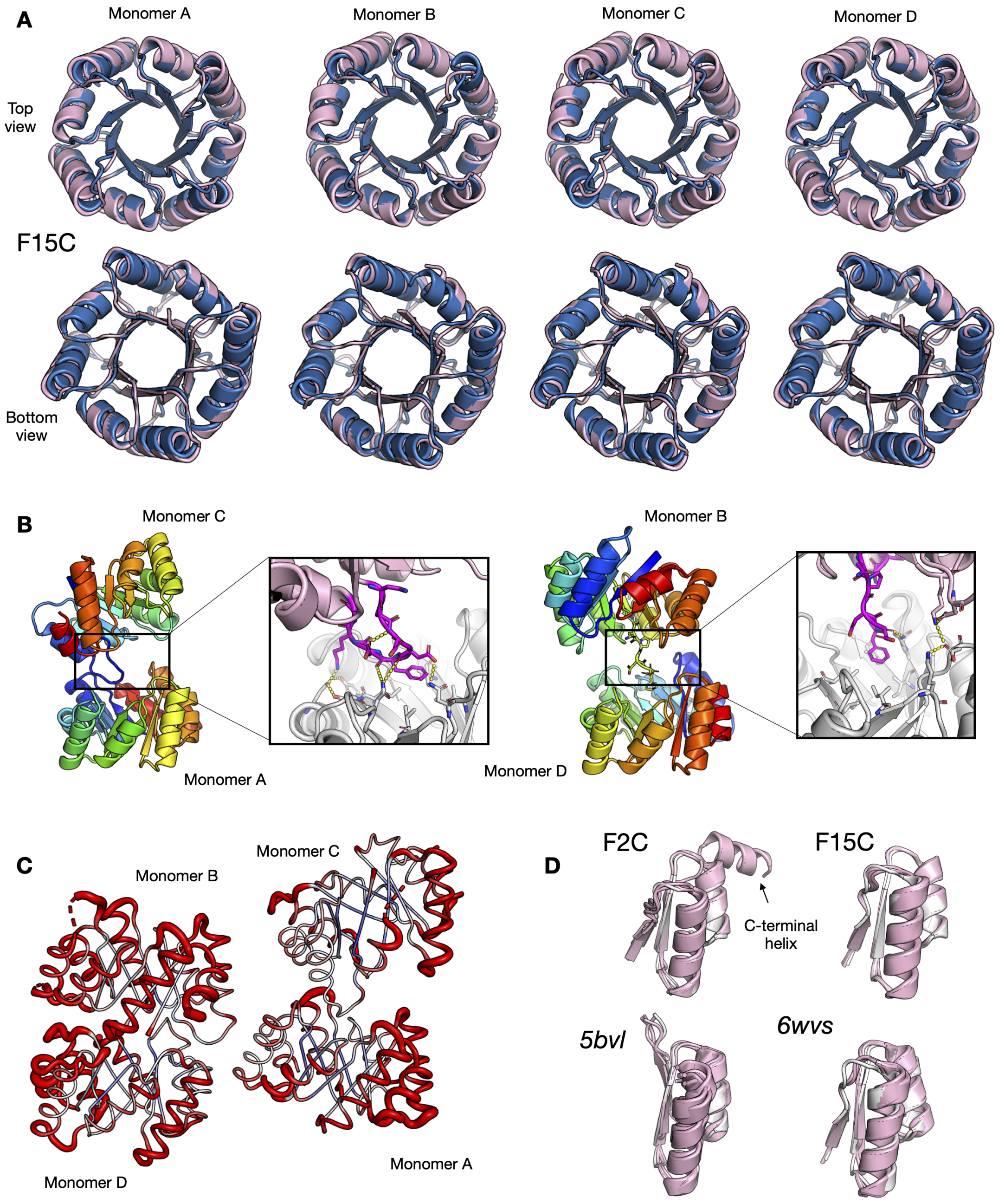

Supplementary Figure 19: Additional crystal structure data for TIM-barrel F15C. **(A)** Overlay with design model (blue) for F15C (pink) for four monomers in asymmetric unit of crystal. **(B)** For two monomers of the tetrameric asymmetric unit of F15C (Monomers C, B), the β-α loop to the long helix for one subunit dislodges to interact with the top of the barrel of an adjacent monomer (Monomers A, D) **(C)** Tetrameric asymmetric unit for F15C crystal, colored by B-factor. **(D)** Symmetric units (pink) overlaid with design template (gray).

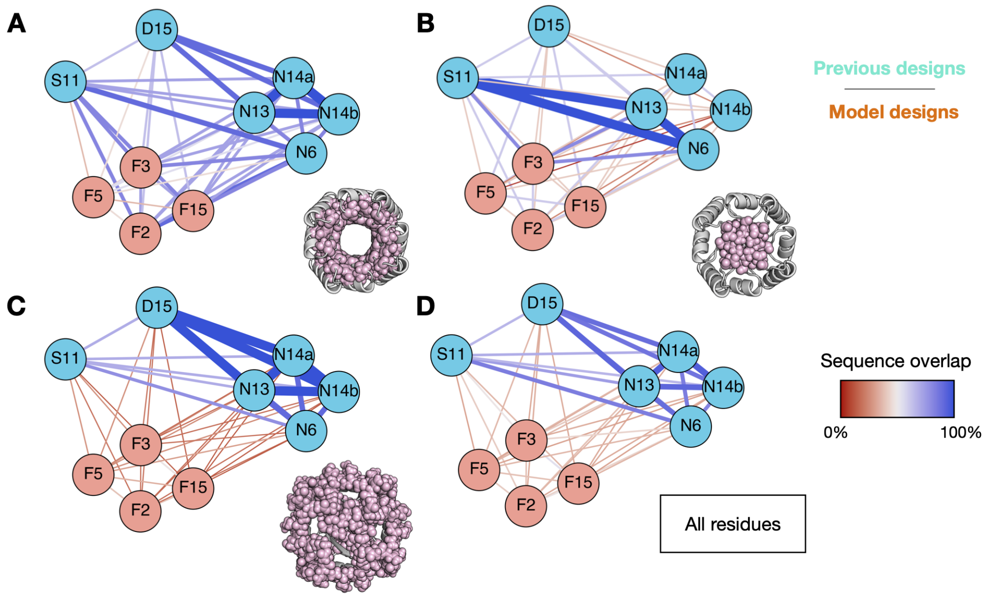

Supplementary Figure 20: Sequence percent identity between TIM-barrel model designs and previously characterized sequences. Sequence percent identity between subunits for model TIM-barrel designs (orange) and previously characterized sequences for the same scaffold (blue), including sTIM-11 (*5bvl*, S11) (*32*), DeNovoTIM15 (*6wvs*, D15) (*33*), and DeNovoTIMs (N6, N13, N14a, N14b) (*34*). N14a and N14b are two quarters of the two-fold symmetric DeNovoTIM14. Specific overlaps shown for the region between (**A**) the helices and the barrel, (**B**) the inner part of the barrel, (**C**) exposed regions, and (**D**) all residues.

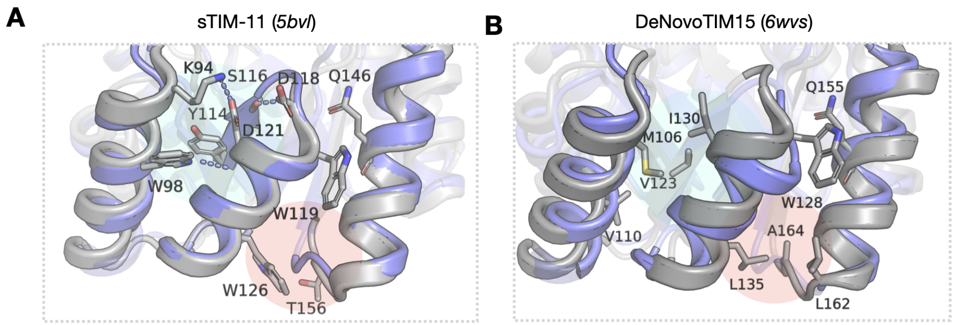

Supplementary Figure 21: Sequence features for previously characterized TIM-barrel designs. Features highlighted for the symmetric subunit near the top of the TIM-barrel (cyan) and the helix interface between symmetric subunits (orange) for previously characterized designs (**A**) sTIM-11 and (**B**) DeNovoTIM15 with sTIM-11 Rosetta model (design scaffold) overlaid in purple.

Supplementary Tables

Backbone	Design	Filters and selection procedure
1cc8	Filters	capping residue percentage -- 100%; packstat >= 0.55; packstat post-RosettaRelax >= 0.55
	d1	3rd lowest model negative PLL on constrained relaxed backbone
	d2	lowest model negative PLL on constrained relaxed backbone
	d3	lowest model negative PLL on crystal structure
	d4	2nd lowest model negative PLL on constrained relaxed backbone
3mx7	Filters	packstat >= 0.55; packstat post-RosettaRelax >= 0.55; sidechain buried unsats <=2
	d1	2nd lowest model negative PLL on constrained relaxed backbone
	d2	lowest model negative PLL on constrained relaxed backbone
	d3	lowest model negative PLL on crystal structure
	d4	2nd lowest model negative PLL on crystal structure
1acf	Filters	packstat >= 0.55; packstat post-RosettaRelax >= 0.55; backbone buried unsats <=10
	d1	ranked 15th in model PLL on constrained relaxed backbone. Has designed disulfide. Negative control for PLL ranking.
	d2	lowest model negative PLL on constrained relaxed backbone without packstat filters (packstat -- 0.54, 0.487 post RosettaRelax)
	d3	lowest model negative PLL on crystal structure
	d4	lowest model negative PLL on constrained relaxed backbone
1bkr	Filters	capping residue percentage -- 100%; packstat >= 0.55; packstat post-RosettaRelax >= 0.55
	d1	ranked 12th in model PLL on constrained relaxed backbone. Negative control for PLL ranking.
	d2	4th lowest model negative PLL on constrained relaxed backbone and highest post-RosettaRelax packstat among top 4 designs ranked by model PLL
	d3	2nd lowest model negative PLL on crystal structure without packstat filters (packstat -- 0.507, 0.562 post RosettaRelax)
	d4	3rd lowest model negative PLL on crystal structure and highest post-RosettaRelax packstat among top 3 designs ranked by model PLL

Supplementary Table 1: Selection of native test case designs for further characterization. Unless otherwise stated, designs were selected based on model PLL rank after applying specified filters across 50 designs. Designs were done on crystal structures as well as crystal structures relaxed under the Rosetta energy function but constrained to the starting coordinates (constrained relaxed backbones). Top sequences highlighted in main paper in Fig. 2I-J are *1acf d3*, *1bkr d2*, *1cc8 d2*, and *3mx7 d4*.

	1	2	3	4	5	6	7	8	9	10	11	12	13	14	15	16	17	18	19	20	21	22	23	24	25
1acf	S	W	Q	T	Y	V	D	T	N	L	V	G	T	G	A	V	T	Q	A	A	I	L	G	L	D
1acf d1	S	D	D	E	I	V	L	K	D	L	V	A	T	G	K	V	T	K	A	A	L	M	T	L	D
1acf d2	D	W	E	K	I	V	Q	E	R	L	V	A	S	G	K	V	L	K	A	A	I	L	S	L	D
1acf d3	S	A	Q	E	I	V	D	K	R	L	V	A	T	G	K	V	K	K	A	A	M	L	K	L	S
1acf d4	N	D	E	D	K	V	I	K	K	L	V	A	S	G	N	V	K	K	A	A	I	L	R	L	D

	26	27	28	29	30	31	32	33	34	35	36	37	38	39	40	41	42	43	44	45	46	47	48	49	50
1acf	G	N	T	W	A	T	S	A	G	F	A	V	T	P	A	Q	G	T	T	L	A	G	A	F	N
1acf d1	G	N	L	R	A	K	S	P	G	F	S	V	T	L	E	E	A	R	A	L	A	R	F	F	E
1acf d2	G	K	V	L	A	A	S	P	G	F	T	V	T	E	E	E	A	K	Q	L	A	K	A	F	D
1acf d3	G	D	V	I	A	A	A	P	G	F	N	V	T	P	E	E	A	K	K	L	A	A	A	F	N
1acf d4	G	S	V	K	A	A	A	P	G	F	N	V	T	P	E	Q	A	K	R	L	A	A	A	F	D

	51	52	53	54	55	56	57	58	59	60	61	62	63	64	65	66	67	68	69	70	71	72	73	74	75
1acf	N	A	D	A	I	R	A	G	G	F	D	L	A	G	V	H	Y	V	T	L	R	A	D	D	R
1acf d1	D	C	A	E	A	E	R	K	G	V	Y	L	N	G	E	K	F	E	I	E	K	C	S	S	V
1acf d2	D	G	S	E	L	R	S	K	G	A	K	L	N	G	E	L	Y	Q	V	K	E	L	S	A	D
1acf d3	D	G	S	F	A	K	K	Q	G	L	T	L	D	G	T	V	F	K	V	E	K	L	T	K	D
1acf d4	D	G	A	Y	V	K	K	N	G	L	E	L	D	G	T	V	Y	K	V	D	K	I	T	K	D

	76	77	78	79	80	81	82	83	84	85	86	87	88	89	90	91	92	93	94	95	96	97	98	99	100
1acf	S	I	Y	G	K	K	G	S	S	G	V	I	T	V	K	T	S	K	A	I	L	V	G	V	Y
1acf d1	E	I	I	L	K	K	G	D	T	R	A	Y	V	F	K	C	K	K	V	V	I	I	A	I	N
1acf d2	K	I	T	F	E	K	G	K	K	H	A	E	A	Y	R	T	N	K	I	I	I	V	A	V	A
1acf d3	E	I	I	F	K	S	G	D	S	A	A	V	A	R	K	L	D	K	V	I	L	I	A	L	V
1acf d4	I	I	E	L	R	A	G	Q	T	G	A	V	C	V	K	T	N	K	E	I	I	I	A	I	Y

	101	102	103	104	105	106	107	108	109	110	111	112	113	114	115	116	117	118	119	120	121	122	123	124	125
1acf	N	E	K	I	Q	P	G	T	A	A	N	V	V	E	K	L	A	D	Y	L	I	G	Q	G	F
1acf d1	T	N	K	H	D	P	V	E	T	K	K	A	V	E	E	K	R	I	E	L	E	A	T	G	E
1acf d2	D	E	K	R	N	P	N	E	T	A	E	K	A	K	N	L	D	R	E	L	K	S	E	G	L
1acf d3	D	G	K	I	N	L	A	E	A	I	A	Q	V	S	A	L	A	D	E	L	K	K	E	G	K
1acf d4	D	D	K	L	D	R	K	K	T	I	E	K	A	K	S	L	N	E	E	M	T	A	E	G	L

Supplementary Table 2: Sequence alignment of designs on *1acf* backbone selected for experimental characterization. Mutations relative to native sequence are highlighted in red.

	1	2	3	4	5	6	7	8	9	10	11	12	13	14	15	16	17	18	19	20	21	22	23	24	25
1bkr	K	S	A	K	D	A	L	L	L	W	C	Q	M	K	T	A	G	Y	P	N	V	N	I	H	N
1bkr d1	E	K	A	V	E	E	L	R	Q	W	V	R	S	V	V	Q	G	F	P	N	V	D	F	K	D
1bkr d2	G	V	A	K	E	E	L	I	Q	W	V	R	K	V	I	A	G	Y	P	N	C	T	F	S	S
1bkr d3	S	A	A	D	Q	A	L	L	D	W	I	R	K	V	V	E	G	F	P	N	V	D	V	T	D
1bkr d4	P	E	E	K	Q	K	L	L	D	W	I	K	R	M	T	A	G	F	P	N	V	N	I	E	S

	26	27	28	29	30	31	32	33	34	35	36	37	38	39	40	41	42	43	44	45	46	47	48	49	50
1bkr	F	T	T	S	W	R	D	G	M	A	F	N	A	L	I	H	K	H	R	P	D	L	I	D	F
1bkr d1	F	T	T	S	C	S	N	G	A	V	F	R	A	I	I	H	K	Y	R	P	E	L	I	D	F
1bkr d2	A	T	Q	S	V	R	L	G	L	V	L	C	A	I	V	H	K	Y	R	P	D	L	I	D	F
1bkr d3	L	T	T	P	I	K	D	G	M	T	L	N	A	I	I	K	K	Y	H	P	S	L	I	D	Y
1bkr d4	L	R	E	S	F	S	D	G	M	A	F	C	A	L	I	Y	A	Y	R	P	D	L	L	K	F

	51	52	53	54	55	56	57	58	59	60	61	62	63	64	65	66	67	68	69	70	71	72	73	74	75
1bkr	D	K	L	K	K	S	N	A	H	Y	N	L	Q	N	A	F	N	L	A	E	Q	H	L	G	L
1bkr d1	S	K	F	K	P	E	T	A	K	E	A	I	E	D	A	M	R	L	A	E	E	K	L	G	I
1bkr d2	A	T	L	K	P	E	D	A	E	R	N	L	Q	L	A	L	D	I	C	E	K	Y	L	G	I
1bkr d3	Q	S	L	K	P	E	N	P	L	Y	N	I	K	L	A	L	E	K	A	E	K	D	L	G	I
1bkr d4	D	K	L	T	P	E	R	P	E	F	N	L	Q	L	A	L	D	I	A	E	K	E	L	G	I

	76	77	78	79	80	81	82	83	84	85	86	87	88	89	90	91	92	93	94	95	96	97	98	99	100
1bkr	T	K	L	L	D	P	E	D	I	S	V	D	H	P	D	E	K	S	I	I	T	Y	V	V	T
1bkr d1	P	K	P	L	D	P	K	E	I	D	K	P	K	P	N	K	E	D	I	I	A	Y	L	K	K
1bkr d2	P	K	K	L	D	P	K	A	L	S	P	Q	E	P	D	E	Q	A	I	L	D	Y	L	Q	M
1bkr d3	P	M	P	L	K	P	E	E	I	I	K	D	Q	P	D	K	Q	K	I	K	E	Y	L	E	I
1bkr d4	P	K	L	I	D	P	K	E	I	S	K	A	K	P	D	E	K	V	I	E	A	Y	L	R	L

	101	102	103	104	105	106	107	108
1bkr	Y	Y	H	Y	F	S	K	M
1bkr d1	L	A	K	Y	F	L	S	S
1bkr d2	L	K	D	K	F	E	A	E
1bkr d3	L	K	E	K	F	E	K	K
1bkr d4	L	E	K	F	M	K	E	G

Supplementary Table 3: Sequence alignment of designs on *1bkr* backbone selected for experimental characterization. Mutations relative to native sequence are highlighted in red.

	1	2	3	4	5	6	7	8	9	10	11	12	13	14	15	16	17	18	19	20	21	22	23	24	25
1cc8	A	E	I	K	H	Y	Q	F	N	V	V	M	T	C	S	G	C	S	G	A	V	N	K	V	L
1cc8 d1	G	K	V	K	I	Y	E	F	E	V	V	M	K	S	E	E	D	A	K	K	V	R	E	V	L
1cc8 d2	N	E	W	K	E	Y	V	F	K	V	V	M	K	S	P	E	D	A	K	K	L	Q	I	V	L
1cc8 d3	G	E	V	Q	K	L	T	F	V	V	K	M	T	S	P	E	D	A	K	K	L	R	D	V	L
1cc8 d4	G	E	T	K	Y	Y	E	F	K	V	D	M	K	D	D	E	D	A	K	K	V	Q	D	V	L

	26	27	28	29	30	31	32	33	34	35	36	37	38	39	40	41	42	43	44	45	46	47	48	49	50
1cc8	T	K	L	E	P	D	V	S	K	I	D	I	S	L	E	K	Q	L	V	D	V	Y	T	T	L
1cc8 d1	E	S	M	R	P	K	I	K	E	V	E	I	D	L	S	K	N	Q	V	R	V	K	S	T	L
1cc8 d2	D	K	L	R	P	E	V	K	K	T	E	I	D	L	T	K	N	E	L	K	V	Y	S	R	I
1cc8 d3	E	S	L	K	P	Q	V	K	E	I	R	I	K	L	D	K	Q	E	L	E	V	Y	T	T	L
1cc8 d4	D	S	M	K	P	K	V	K	E	V	R	I	D	K	S	K	N	L	V	S	V	F	S	T	L

	51	52	53	54	55	56	57	58	59	60	61	62	63	64	65	66	67	68	69	70	71	72
1cc8	P	Y	D	F	I	L	E	K	I	K	K	T	G	K	E	V	R	S	G	K	Q	L
1cc8 d1	P	F	E	E	I	L	E	K	I	K	K	T	G	I	P	V	L	S	A	K	E	L
1cc8 d2	D	F	E	T	L	K	K	L	L	E	E	T	G	I	K	L	E	S	A	Y	E	K
1cc8 d3	P	Y	E	L	I	E	K	K	I	K	E	T	G	I	E	V	L	S	A	K	K	D
1cc8 d4	P	L	E	E	I	K	K	R	I	E	E	T	G	I	P	V	F	S	W	K	E	L

Supplementary Table 4: Sequence alignment of designs on *1cc8* backbone selected for experimental characterization. Mutations relative to native sequence are highlighted in red.

	1	2	3	4	5	6	7	8	9	10	11	12	13	14	15	16	17	18	19	20	21	22	23	24	25
3mx7	M	T	D	L	V	A	V	W	D	V	A	L	S	D	G	V	H	K	I	E	F	E	H	G	T
3mx7 d1	M	P	D	K	V	A	K	W	V	V	K	L	P	K	K	T	Y	V	I	E	L	E	F	N	P
3mx7 d2	R	K	Y	E	V	A	V	F	K	V	R	L	S	D	K	D	H	E	V	R	I	E	F	D	T
3mx7 d3	L	P	K	L	V	A	K	I	D	V	K	L	A	G	A	T	F	T	I	E	L	E	F	D	P
3mx7 d4	K	E	I	L	K	A	V	F	E	V	E	L	P	G	S	S	Y	K	I	E	L	K	L	D	L

	26	27	28	29	30	31	32	33	34	35	36	37	38	39	40	41	42	43	44	45	46	47	48	49	50
3mx7	T	S	G	K	R	V	V	Y	V	D	G	K	E	E	I	R	K	E	W	M	F	K	L	V	G
3mx7 d1	D	T	G	R	I	K	V	L	V	D	G	K	E	V	I	H	E	A	K	S	D	T	R	E	K
3mx7 d2	K	S	G	L	I	E	V	Y	V	D	G	T	L	V	I	K	I	P	A	S	A	T	K	P	Q
3mx7 d3	E	T	G	E	I	K	V	Y	V	D	G	K	L	V	Y	E	R	K	A	G	K	S	R	P	E
3mx7 d4	E	T	G	K	I	E	V	Y	V	D	G	T	L	K	I	K	I	E	H	R	A	S	L	P	S

	51	52	53	54	55	56	57	58	59	60	61	62	63	64	65	66	67	68	69	70	71	72	73	74	75
3mx7	K	E	T	F	Y	V	G	A	A	K	T	K	A	T	I	N	I	D	A	I	S	G	F	A	Y
3mx7 d1	S	V	T	F	K	V	G	E	D	E	V	P	A	T	I	F	I	K	P	V	P	G	G	T	L
3mx7 d2	S	V	R	F	L	I	G	K	D	K	R	P	A	T	V	L	I	K	P	I	P	G	G	K	Y
3mx7 d3	E	I	K	F	E	V	G	P	E	K	V	P	V	T	V	R	I	R	E	I	A	G	D	K	F
3mx7 d4	S	V	S	F	E	V	G	P	E	K	V	P	A	T	L	T	I	T	P	I	D	G	G	K	Y

	76	77	78	79	80	81	82	83	84	85	86	87	88	89	90
3mx7	E	Y	T	L	E	I	N	G	K	S	L	K	K	Y	M
3mx7 d1	T	F	T	L	V	I	D	G	K	S	E	T	K	S	K
3mx7 d2	T	F	T	L	I	I	D	G	K	S	K	T	E	G	K
3mx7 d3	R	F	E	F	I	I	N	G	T	S	K	E	F	S	Q
3mx7 d4	A	I	T	L	T	I	N	G	K	S	K	T	A	S	E

Supplementary Table 5: Sequence alignment of designs on *3mx7* backbone selected for experimental characterization. Mutations relative to native sequence are highlighted in red.

Design	Sequence rank for metrics of interest across 50 designs												
	Post-design metrics					Post-RosettaRelax metrics						Psipred cross entropy	
	**model PLL**	BB buried unsats	Exposed hydrophobics	Packstat	SC buried unsats	**model PLL**	BB buried unsats	Exposed hydrophobics	Packstat	CA RMSD	SC buried unsats	MSA	Single seq
**F1**	35	23	28	34	18	**2**	**2**	32	**2**	**3**	30	18	25
**F2**	12	**5**	34	32	**7**	**6**	**1**	34	24	**10**	30	43	**8**
**F15**	**5**	**1**	**8**	24	**5**	13	40	**1**	**6**	45	41	**5**	37
**F3**	**8**	23	27	47	18	18	**4**	23	45	37	20	19	27
**F14**	18	**3**	43	33	23	34	22	38	13	28	**1**	45	19
**F5**	23	34	42	46	29	**4**	30	43	14	13	30	27	16
**F4**	16	31	47	**3**	37	31	24	47	20	16	38	**1**	**3**
**F8**	38	**5**	35	22	45	42	40	35	**1**	47	40	40	47
**F11**	46	38	23	43	23	26	47	25	15	**8**	**1**	23	42
**F6**	**3**	**4**	**4**	37	**1**	20	**6**	**4**	21	34	**1**	**4**	**1**
**F10**	**6**	27	14	12	38	**1**	28	18	**8**	11	38	47	32
**F9**	22	23	**9**	**2**	29	17	15	**6**	**7**	41	30	14	**7**
**F7**	**1**	**9**	**4**	19	9	32	24	**8**	31	32	14	20	22

Supplementary Table 6: TIM-barrel sequence selection – metrics-based selection of 13 designs from initial 50 after filtering by packstat ≥ 0.55 pre- and post-RosettaRelax and 100% occurrence of N-terminal helical capping residues. For each metric, the corresponding rank among 50 designs is given. Metrics used to select a particular sequence are highlighted in red. Secondary metrics of interest are bolded. For instance, F7 has high rank in terms of model PLL but also ranks highly in terms of exposed hydrophobics score. (Blue rows) Crystal structures. (Blue, green rows) Folded designs. (Orange rows) Tested designs that do not appear folded or are not monodisperse based on SEC.

Construct	Total structure score	Structural features and scores											
		R13 feature penalty	Buried polar penalty	Buried polar residue	Lumen score	Lumen feature	Asp1 score	Bottom pack score	Bottom pack feature	Helix packing score	Helix packing feature	Gln16/Glu16 score	Gln16/Glu16 feature
**F1**	**21**	0	0		4	Y30	4	4	W23	5	I14, W35	4	Q16
**F2**	**18**	0	0		4	Y30	4	4	Y23, L42	2	I14	4	Q16
**F15**	**18**	0	0		4	Y30	4	3	H23, E42	4	F10, V14, A35	3	E16
**F3**	**16**	0	0		3	I30	4	2	Q42	5	F10, V14, Y35	2	E16
**F14**	**15**	0	0		3	I30	4	2	E42	3	V14, A35	3	E16
**F5**	**15**	4	0		2	V30	4	4	Y23, L42	5	I14, W35	4	Q16
**F4**	**14**	4	0		3	I30	4	4	W23	3	I14, A35	4	Q16
**F8**	**14**	0	4	H30	3	H30	4	4	Y23, L42	4	I14, A35, L39	3	E16
**F11**	**12**	0	0		4	Y30	0	4	Y23, L42	2	A35, I39	2	L16
**F6**	**10**	0	0		3	I30	4	0	R42	2	I14	1	M16
**F10**	**8**	4	4	E30	1	E30	4	4	Y23, L42	4	I14, A35, I39	3	E16
**F9**	**7**	0	0		3	I30	0	1	I42	3	V14, A35	0	N16
**F7**	**6**	0	4	K13	3	I30	0	2	E42	3	I14, A35	2	E16

**R13 feature**	-4 for R13
**Buried polar**	-4 for buried polar residues
**Lumen score**	+4 points for Y30 or F30, +3 for I30 or H30, +2 for V30
**Asp1 score**	+4 for Asp1
**Bottom packing score**	Packing for region between symmetric subunits at bottom of structure. +4 for W23, Y23/L42, +3 for H23/E42, +2 for Q42 or E42, +1 for I42
**Helix packing**	Packing between the helices near the top/middle of the structure. Scores below. +2 for I14 or V14, +2 for F10, +3 for W35, +1 for A35, +2 for Y35, +1 for I39 or L39
**Gln16/Glu16 score**	Polar contact linking top of helices. +4 points for Q16, +3 for E16, +2 for L16, +1 for M16, +0 for N16

Supplementary Table 7: TIM-barrel sequence selection – structural feature-based selection. Selection of top 8 designs from 13 based on simple criteria for a set of structural features. (Top) Total scores, scores across different critera, and corresponding features for criteria. (Bottom) Description of criteria and scoring methodology. (Blue rows) Crystal structures. (Blue, green rows) Folded designs. (Orange rows) Tested designs that do not appear folded or are not monodisperse based on SEC.

	1	2	3	4	5	6	7	8	9	10	11	12	13	14	15	16	17	18	19	20	21	22	23
sTIM11	D	I	L	I	V/C	D	A	T	D	K	D	E	A	W	K	Q/C	V	E	Q	L	R	R	E
DeNovoTIM15	D	I	L	I	V	N	A	T	D	**V**	D	E	**M**	**L**	K	Q	V	E	**I**	L	R	R	**L**
NovoTIM6	D	I	L	I	V	D	A	T	D	**V**	D	E	A	W	K	Q	V	E	**I**	L	R	R	**L**
NovoTIM13	D	I	L	I	V	D	A	T	D	**V**	D	E	**M**	**L**	K	Q	V	E	**I**	L	R	R	**L**
NovoTIM14a	D	I	L	I	V	D	A	T	D	**V**	D	E	**M**	**L**	K	Q	V	E	**I**	L	R	R	**L**
NovoTIM14b	D	**V**	L	I	V	D	A	T	D	**V**	D	E	**M**	**L**	K	Q	V	E	**I**	L	R	R	**L**
F2	D	I	**A**	I	V	D	A	**D**	**N**	**P**	**A**	**D**	A	**I**	**Q**	Q	V	**K**	**D**	L	R	**K**	**Y**
F15	D	I	L	I	C->V	**N**	**P**	**D**	D	**F**	**E**	**K**	**G**	**V**	**E**	**E**	V	**K**	**E**	L	**K**	R	**H**
F5	D	I	**V**	I	**M**	**N**	**H**	**D**	D	**L**	**A**	E	**R**	**I**	K	Q	V	**Q**	**R**	L	**K**	**E**	**Y**
F3	D	I	L	I	V	D	**H**	**A**	D	**F**	D	**K**	A	**V**	**E**	**E**	V	**K**	Q	L	**K**	**K**	E

	24	25	26	27	28	29	30	31	32	33	34	35	36	37	38	39	40	41	42	43	44	45	46
sTIM11	G	A	T	Q	I	A	Y	R	S	D	D	W	R	D	L	K	E	A	W	K	K	G	A
DeNovoTIM15	G	A	**K**	Q	I	A	**V**	**V**	S	D	D	W	R	**I**	L	**Q**	E	A	**L**	K	K	G	**G**
NovoTIM6	G	A	**K**	Q	I	A	Y	R	S	D	D	W	R	D	L	**Q**	E	A	**L**	K	K	G	A
NovoTIM13	G	A	**K**	Q	I	A	**V**	R	S	D	D	W	R	**I**	L	**Q**	E	A	**L**	K	K	G	A
NovoTIM14a	G	A	**K**	Q	I	**W**	**V**	**I**	S	D	D	W	R	**I**	L	**Q**	E	A	**L**	K	K	G	A
NovoTIM14b	G	A	**K**	Q	I	**V**	**V**	**I**	S	D	D	W	R	**I**	L	**Q**	E	A	**L**	K	K	G	A
F2	G	A	**K**	**L**	I	A	Y	**K**	S	**K**	**S**	**S**	**E**	**E**	L	K	**L**	A	**L**	K	**A**	G	A
F15	G	A	**K**	**I**	I	A	Y	**I**	S	**K**	**S**	**A**	**E**	**E**	L	K	**K**	A	**E**	K	**A**	G	A
F5	G	A	**K**	**Y**	I	A	**V**	**L**	**T**	**K**	**N**	W	**E**	D	**G**	**V**	**K**	A	**L**	K	**A**	G	A
F3	G	A	**K**	**Y**	I	A	**I**	R	S	D	D	**Y**	**K**	**K**	L	K	**K**	A	**Q**	**D**	**A**	G	A

Supplementary Table 8: Sequence alignment of symmetric subunits for previously characterized four-fold or nearly four-fold symmetric TIM barrel designs (sTIM-11, DeNovoTIM15, DeNovoTIMs) with model designs (FX). N14a and N14b are two quarters of the two-fold symmetric DeNovoTIM14. Mutations relative to sTIM-11 highlighted in red. Blue columns indicate positions where the previous designs are convergent and one or more model designs differ. Green columns indicate positions where one or more model designs differ from sTIM-11, as does a previously characterized design.

Crystallographic parameters, data collection and refinement statistics

	F2C	F15C	F2N
**Crystallographic parameters**<br><br>Space group<br>Unit-cell dimensions (Å)<br>Asymmetric unit	$P2_12_12_1$<br>44.75, 55.73, 78.72<br>monomer	$P2_1$<br>44.23, 103.57, 80.94;<br>β=90.4°<br>tetramer	$P2_12_12_1$<br>44.13, 48.72,<br>75.58<br>monomer
**Data collection statistics**<br><br>Resolution limits (Å)<br>No: of observed reflections<br>No: of unique reflections<br>Completeness<br>overall/outer shell<br>CC1/2 (overall/outer shell)<br>R_{sym}[a] (%)<br>overall/outer shell & os I/σ	39.4 – 1.46<br>(1.50-1.46)<br>446777<br>34906<br><br>99.8/99.8<br>99.9/71.8<br><br>7.5/171.1 & 1.6	38.93-1.90 (1.95-1.90)<br>792464<br>56979<br><br>99.2/99.8<br>99.7/74.3<br><br>14.7/160.4 & 1.5	38.93-1.58<br>(1.62-1.58)<br>808181<br>24478<br><br>99.9/99.9<br>100/81.8<br><br>5.1/219.0 & 1.4
**Refinement statistics**<br><br>Resolution limits<br>Number of reflections/%<br>(|F|>2σ|F|)<br>Reflections used for R_{free}<br>Rfactor[b] (%)<br>R_{free} (%)<br><br>Model contents/average B(Å^2)<br>Protein atoms<br>Ions<br>Water molecules<br><br>RMS deviations<br>Bond length (Å)<br>Bond angle (°)<br>Ramachandran (favored/<br>outliers)<br>PDB code	39.4-1.46<br>33097/99.8<br><br>1742<br>17.3<br>21.9<br><br>1473/28.4<br>20/46.3<br>178/36.3<br><br>0.010<br>1.432<br>99.0/1<br><br>7MCC	38.93-1.90<br>54130/99.2<br><br>2849<br>17.7<br>25.7<br><br>5684/52.7<br>0<br>144/47.2<br><br>0.006<br>1.35<br>97.8/4<br><br>7MCD	37.8-1.58<br>23254/99.9<br><br>1224<br>17.5<br>23.8<br><br>1322/53.4<br>0<br>30/49.0<br><br>0.010<br>1.54<br>96.0/1<br><br>7SMJ

[a] $R_{sym} = \Sigma|I_{avg}-I_i|/\Sigma I_i$
[b] R factor = $\Sigma|F_p - F_{pcalc}|/\Sigma F_p$, where F_p and F_{pcalc} are the observed and calculated structure factors; R_{free} is calculated with 5% of the data.

Supplementary Table 9: Crystallographic parameters, data collection, and refinement statistics for TIM-barrel crystal structures.

Structure	Unit 1	Unit 2	Unit 3	Unit 4	All (aligned)	All
F2C	0.831	0.694	0.612	6.272	0.769	3.345
F2N	1.448	1.123	0.567	0.811	0.868	1.351
F15C A	0.827	0.685	0.549	0.602	0.757	0.879
F15C B	0.995	0.5	2.367	1.085	0.741	1.184
F15C C	1.607	0.575	0.591	0.931	0.878	1.27
F15C D	1.027	0.622	0.665	0.613	0.826	0.987
5bvl	1.164	0.779	0.746	0.831	0.679	1.256
6wvs	1.178	1.466	1.576	1.49	1.407	1.541

Supplementary Table 10: TIM-barrel crystal structure alpha-carbon (CA) RMSD (Å) to design scaffold prepared with sTIM-11 sequence. CA RMSD for each symmetric unit, all aligned alpha-carbons, and all alpha-carbons. Data for F2C, four F15C monomers, *5bvl* (*32*), and *6wvs* (*33*).
