# Supplementary material for: Protein sequence design with a learned potential
Source: Nat Commun. 2022 Feb 8;13:746. doi: 10.1038/s41467-022-28313-9 (PMC8826426; doi:10.1038/s41467-022-28313-9)
Supplement: Supplementary file 3 — Description of Additional Supplementary Files [file 41467_2022_28313_MOESM3_ESM.pdf]

Description of Additional Supplementary Files

File Name: Supplementary Data 1
Description: Training information, including model architecture, excluded ions/ligands, train/test CATH topology classes, and train/test CATH domains.

File Name: Supplementary Data 2
Description: Native test case analysis data including rotamer recovery data, sequence design metrics, amino acid distribution data post-design, decoy ranking, and runtime data.

File Name: Supplementary Data 3
Description: TIM-barrel design data, including design metrics and selected sequence IDs.

File Name: Supplementary Movie 1
Description: Examples of model-guided rotamer repacking on native backbone test cases.

File Name: Supplementary Movie 2
Description: Examples of model-guided sequence and rotamer design via annealing on native backbone test cases and de novo TIM-barrel scaffold.
