# Supplementary material for: Protein sequence design with a learned potential
Source: Nat Commun. 2022 Feb 8;13:746. doi: 10.1038/s41467-022-28313-9 (PMC8826426; doi:10.1038/s41467-022-28313-9)
Supplement: Supplementary file 9 — Reporting Summary [file 41467_2022_28313_MOESM9_ESM.pdf]

nature portfolio

Corresponding author(s): Po-Ssu Huang

Last updated by author(s): Dec 18, 2021

Reporting Summary

Nature Portfolio wishes to improve the reproducibility of the work that we publish. This form provides structure for consistency and transparency in reporting. For further information on Nature Portfolio policies, see our Editorial Policies and the Editorial Policy Checklist.

Statistics

For all statistical analyses, confirm that the following items are present in the figure legend, table legend, main text, or Methods section.

n/a	Confirmed	
☐	☒	The exact sample size (*n*) for each experimental group/condition, given as a discrete number and unit of measurement
☒	☐	A statement on whether measurements were taken from distinct samples or whether the same sample was measured repeatedly
☒	☐	The statistical test(s) used AND whether they are one- or two-sided<br>*Only common tests should be described solely by name; describe more complex techniques in the Methods section.*
☒	☐	A description of all covariates tested
☒	☐	A description of any assumptions or corrections, such as tests of normality and adjustment for multiple comparisons
☐	☒	A full description of the statistical parameters including central tendency (e.g. means) or other basic estimates (e.g. regression coefficient) AND variation (e.g. standard deviation) or associated estimates of uncertainty (e.g. confidence intervals)
☒	☐	For null hypothesis testing, the test statistic (e.g. *F*, *t*, *r*) with confidence intervals, effect sizes, degrees of freedom and *P* value noted<br>*Give P values as exact values whenever suitable.*
☒	☐	For Bayesian analysis, information on the choice of priors and Markov chain Monte Carlo settings
☒	☐	For hierarchical and complex designs, identification of the appropriate level for tests and full reporting of outcomes
☒	☐	Estimates of effect sizes (e.g. Cohen's *d*, Pearson's *r*), indicating how they were calculated

Our web collection on statistics for biologists contains articles on many of the points above.

Software and code

Policy information about availability of computer code

Data collection	Code to generate dataset, train models, and run design algorithm available at https://github.com/ProteinDesignLab/protein_seq_des. PyTorch 1.1.0 with CUDA 9.0 used to train models. Commands to reproduce design runs in paper given in Supplementary Datasets S2 and S3.
Data analysis	Rosetta 3.12 and PyRosetta4 used for Rosetta computational design experiments. All design runs are reproducible using our open-source code, with commands to reproduce design runs in paper given in Supplementary Datasets S2 and S3. XDS (v Jan 31, 2020), COOT (v 0.9.6), and REFMAC (v 5.8.0267) used for crystallography.

For manuscripts utilizing custom algorithms or software that are central to the research but not yet described in published literature, software must be made available to editors and reviewers. We strongly encourage code deposition in a community repository (e.g. GitHub). See the Nature Portfolio guidelines for submitting code & software for further information.

Data

Policy information about availability of data

All manuscripts must include a data availability statement. This statement should provide the following information, where applicable:

- Accession codes, unique identifiers, or web links for publicly available datasets
- A description of any restrictions on data availability
- For clinical datasets or third party data, please ensure that the statement adheres to our policy

All data generated in this study are included in the manuscript, supplementary data files, and links listed in our open-source repository. Training data is publicly available from the PDB (Protein Data Bank) but also provided in prepared form at this link: https://console.cloud.google.com/storage/browser/seq-des-data. Pre-trained models are available at https://drive.google.com/file/d/1X66RLbaA2-qTlJLlG9TI53cao8gaKnEt/view?usp=sharing.

Field-specific reporting

Please select the one below that is the best fit for your research. If you are not sure, read the appropriate sections before making your selection.

[x] Life sciences [ ] Behavioural & social sciences [ ] Ecological, evolutionary & environmental sciences

For a reference copy of the document with all sections, see nature.com/documents/nr-reporting-summary-flat.pdf

Life sciences study design

All studies must disclose on these points even when the disclosure is negative.

Sample size	The number of protein test cases and the number of test cases were selected 1) in order to design enough sequences to fairly evaluate the distribution of scores across a range of computational metrics and 2) taking into account limited bandwidth for purification and analysis of designed proteins.
Data exclusions	No data was excluded.
Replication	All design runs were seeded and are reproducible, with commands provided in the supplement to reproduce designs.
Randomization	Not applicable -- we did not have control groups or randomization in this study.
Blinding	Blinding was not relevant to either the computational method development or the protein purification and analysis portions of this study.

Reporting for specific materials, systems and methods

We require information from authors about some types of materials, experimental systems and methods used in many studies. Here, indicate whether each material, system or method listed is relevant to your study. If you are not sure if a list item applies to your research, read the appropriate section before selecting a response.

Materials & experimental systems

n/a	Involved in the study	
[x]	[ ]	Antibodies
[x]	[ ]	Eukaryotic cell lines
[x]	[ ]	Palaeontology and archaeology
[ ]	[x]	Animals and other organisms
[x]	[ ]	Human research participants
[x]	[ ]	Clinical data
[x]	[ ]	Dual use research of concern

Methods

n/a	Involved in the study	
[x]	[ ]	ChIP-seq
[x]	[ ]	Flow cytometry
[x]	[ ]	MRI-based neuroimaging

Animals and other organisms

Policy information about studies involving animals; ARRIVE guidelines recommended for reporting animal research

Laboratory animals	No laboratory animals involved. E. coli (BL21 DE3 and DH5alpha) used
Wild animals	No wild animals involved.
Field-collected samples	No field-collected samples.
Ethics oversight	No ethical approval or guidance required (no human or animal subjects).

Note that full information on the approval of the study protocol must also be provided in the manuscript.
